# Rapid Adaptation of Cyanobacteria to Environmental Perturbations Is Achieved Through Structural Remodeling of the Proteome

**DOI:** 10.1016/j.mcpro.2025.101443

**Published:** 2025-11-03

**Authors:** Snigdha Sarkar, Elise M. Van Fossen, Xiaolu Li, Tong Zhang, Song Feng, Victoria Prozapas, Ivo Díaz Ludovico, Abdullah D. Shouaib, Chelsea M. Hutchinson-Bunch, Natalie C. Sadler, Isaac K. Attah, Wei-Jun Qian, Margaret S. Cheung, Pavlo Bohutskyi, John T. Melchior

**Affiliations:** 1 Biological Sciences Division, Earth and Biological Sciences Directorate, Pacific Northwest National Laboratory, Richland, Washington, USA; 2 Nuclear Chem Bio Technologies, National Security Directorate, Pacific Northwest National Laboratory, Richland, Washington, USA; 3 Environmental Molecular Sciences Laboratory, Earth and Biological Sciences Directorate, Pacific Northwest National Laboratory, Richland, Washington, USA; 4 Department of Physics, University of Washington, Seattle, Washington, USA; 5 Department of Biological Systems Engineering, Washington State University, Pullman, Washington, USA; 6 Department of Pathology and Laboratory Medicine, University of Cincinnati, Cincinnati, Ohio, USA; 7 Department of Neurology, Oregon Health and Science University, Portland, Oregon, USA

**Keywords:** structural proteomics, redox proteomics, limited proteolysis- based mass spectrometry, thermal proteome profiling, cyanobacteria

## Abstract

Dynamic environments require cyanobacteria to rapidly respond to fluctuating light conditions on timescales faster than transcription-translation processes allow, which is possible through immediate regulation of protein function *via* molecular and conformational adjustments. Traditional abundance-based proteomics cannot capture these rapid structural changes, creating a critical gap in understanding cellular adaptation mechanisms. We hypothesized that application of alternative structural proteomics approaches would enable identification of immediate structural remodeling across the cyanobacterial proteome triggered by environmental perturbations, potentially driving functional adaptations invisible to conventional abundance-based methods. We interrogated three complementary techniques—limited proteolysis-based mass spectrometry, thermal proteome profiling, and redox proteomics—for their capacity to unveil structural reorganization within the model cyanobacterium *Synechococcus elongatus* PCC 7942 during physiologically relevant light transitions. Within 30 min of increased light exposure, we detected structural changes in 753 proteins (limited proteolysis-based mass spectrometry), thermal stability shifts in 600 proteins (thermal proteome profiling), and cysteine oxidation in 1887 sites, while only 145 proteins changed in abundance. All three techniques consistently revealed coordinated remodeling of photosynthetic machinery, ribosomal complexes, and carbon metabolism, exemplified by cytochrome f stabilization modulating electron transport efficiency. Remarkably, <10% of proteins overlapped between methods, demonstrating that each technique captures distinct molecular dimensions of environmental adaptation. This structural proteomics framework demonstrates how alternative techniques can reveal hidden facets of proteome dynamics underlying cellular processes, offering new methodological approaches for understanding environmental responses and informing biotechnological applications.

## Highlights

• Structural proteomics reveal how cyanobacteria adapt to changing light.
• Light shifts trigger remodeling of phycobilisome and electron transport proteins.
• Data integration shows how photosynthesis regulates carbon metabolism pathways.

## Graphical Abstract

**Figure:**
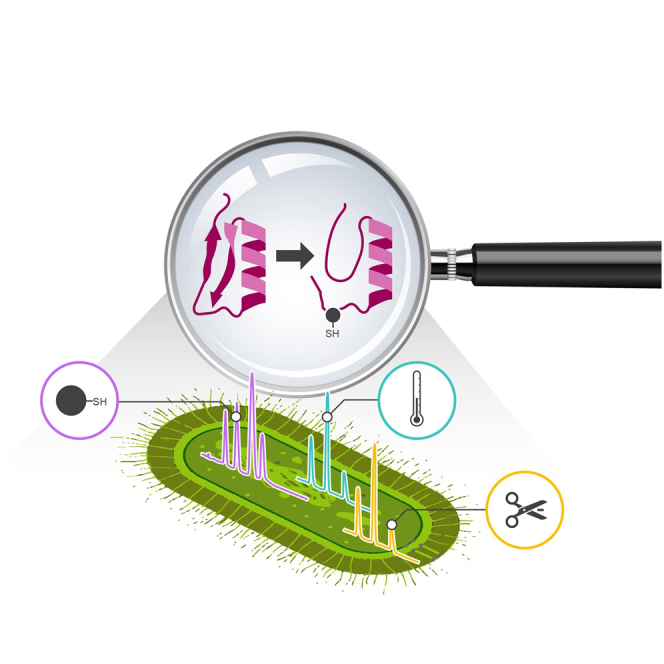

## In Brief

Cyanobacteria adapt swiftly to environmental changes, but the molecular mechanisms remain unclear. Here, using three complementary structural proteomics techniques, the authors reveal extensive proteome-wide remodeling triggered by light shifts. These changes impact assemblies critical for photosynthesis, translation, and carbon metabolism, uncovering a hidden regulatory layer invisible to traditional proteomics. This study demonstrates how integrating structural proteomics provides novel insights into the rapid adaptations driving microbial responses to dynamic environments.

Cyanobacteria are ancient photosynthetic prokaryotes that transformed Earth’s atmosphere through oxygenation, facilitating the emergence of aerobic metabolism and complex life forms (1). These organisms exhibit remarkable metabolic versatility, being capable of primary carbon and nitrogen fixation (2) and the synthesis of diverse bioactive compounds (3). These attributes make them critical players in global biogeochemical cycles and promising candidates for biotechnological applications, including the sustainable production of biofuel precursors, high-value chemicals, as well as bioremediation (4). However, cyanobacteria experience both cyclic and unexpected light fluctuations—from self-shading in dense cultures to variable sunlight exposure—that challenge productivity optimization due to our limited understanding of how they rapidly adapt to such perturbations (5, 6). Elucidating these fundamental adaptation mechanisms will enable rational engineering of cyanobacteria toward increased tolerance to light fluctuations, leading to improved cellular responses and enhanced metabolic outputs.

Phototroph responses to fluctuating light environments occur through sophisticated multilayer regulatory mechanisms that operate across vastly different timescales (7, 8). At the physiological level, cyanobacteria face complex light dynamics in both natural ecosystems and industrial cultivation systems that require immediate molecular responses faster than transcriptional regulation allows (9, 10). In natural environments, cells experience variable light availability due to water column mixing, cloud cover, and diurnal cycles on timescales of minutes to hours. Similarly, in high-density cultivation systems, light gradients develop as cultures reach biotechnologically relevant cell concentrations, creating distinct microenvironments where interior cells face light limitation while outer cells experience light saturation (11). These environmental fluctuations demand immediate cellular responses that occur on timescales incompatible with gene expression changes. Understanding how cells achieve these rapid adaptations is essential for optimizing industrial cyanobacterial systems, where maintaining uniform productivity across cell populations directly impacts process economics (12).

Proteins orchestrate rapid cellular responses to environmental changes, making them critical molecular readouts for understanding how organisms rapidly respond to light perturbations and for defining cellular phenotypes. Current gold-standard systems biology approaches infer cellular function by quantifying changes in global protein abundance, using advanced mass spectrometers (LC-MS) (13, 14). While informative, these measures fail to capture what decades of studies have shown to be key drivers of cellular function—changes in protein structure (15). The characteristic conformational adaptability of proteins through post-translational modifications (PTMs) or interactions with other proteins or metabolites enables rapid responses to changing environments (16, 17), particularly in cyanobacteria, where photosynthesis-based metabolism demands highly dynamic protein structural regulation (18).

Protein structural regulation enables cyanobacteria to integrate light capture efficiency with protective mechanisms, coordinate electron transport chain activity with carbon fixation capacity, and synchronize circadian regulation with metabolic demands (19). Critical protein complexes such as light-harvesting antennae, photosystem reaction centers, carbon concentrating machinery, and protein synthesis apparatus require constant fine-tuning to optimize function under variable light conditions (20, 21). These molecular machines possess inherent structural flexibility that allows rapid functional adjustments through conformational switches, subunit rearrangements, and interaction partner exchanges. Such structural adaptations represent a fundamental regulatory layer that operates independently of protein abundance changes, creating a critical knowledge gap when relying solely on traditional proteomics approaches. This limitation constrains mechanistic understanding of cyanobacterial physiology and hampers rational engineering of optimized strains.

There is a critical need for new high-throughput tools for systems biology analysis to enable better understanding of proteome-wide structural dynamics that define rapid phenotype adjustments in response to environmental perturbations in phototrophs (5). Though traditional high-resolution structural biology tools such as X-ray crystallography, nuclear magnetic resonance, and cryo-electron microscopy offer exquisite insight into protein structure-function relationships, these techniques are labor-intensive and often capture structural details of single, isolated proteins. Recent advances in structural proteomics leverage the high-throughput capability of LC-MS to enable characterization of the structural changes in the global proteome. As part of the Predictive Phenomics Initiative at Pacific Northwest National Laboratory, our team has established a suite of structural proteomics workflows to interrogate the relationship between protein structural remodeling and cellular adaptation. We hypothesized that complementary structural proteomics approaches can reveal extensive proteome remodeling invisible to traditional abundance-based proteomics and enable mechanistic insights into rapid cellular responses to environmental perturbations. Herein, we apply three of these advanced technologies, limited proteolysis-based mass spectrometry (LiP-MS) (22), thermal proteome profiling-based mass spectrometry (TPP-MS) (23), and redox proteomics (24) to *Synechococcus elongatus* PCC 7942 (*S. elongatus)*, a well-characterized cyanobacterium widely used to investigate environmental adaptation mechanisms, develop computational models of metabolism and regulation, and serve as a test chassis for biotechnology research (25). We show the power of these complementary technologies in illuminating functional pathways, key macromolecular assemblies, and individual proteins that modulate *S. elongatus* adaptation to different light conditions, simulating real-world environmental and industrial scenarios.

## Experimental Procedures

### Organism Growth and Maintenance

We performed studies on the cyanobacterial strain *S. elongatus* PCC 7942 (cscB/SPS) (26, 27, 28) which was kindly provided by Daniel Ducat from Michigan State University, East Lansing, MI. *S. elongatus* cultures were maintained at 29 ± 2 °C in 1.2 L Roux culture flasks containing BG-11 medium (0.4–0.6 L) supplemented with (NH_4_)_2_SO_4_ (0.264 g/L), K_2_HPO_4_ (0.174 g/L), yeast nitrogen base without amino acids, (NH_4_)_2_SO_4_ (0.09 g/L, Y1251, Sigma-Aldrich), and Hepes (3 g/L, pH 8.4). We grew and maintained cultures under constant light using Monios-L LED Full Spectrum Grow Lights at an intensity of 200 μmol photons m^-2^ s^-1^ as measured by LI-250A Light Meter with LI-190SA Quantum Sensor (LI-COR, Inc). The culture medium was continuously mixed with a PTFE magnetic stir bar at ∼200 rpm and sparged with N_2_ gas containing ∼2% CO_2_. Cells were diluted regularly (every 3–4 days) to *A*_750_ = 0.2.

### Cyanobacterial Sample Harvest

**Fig. 1:**
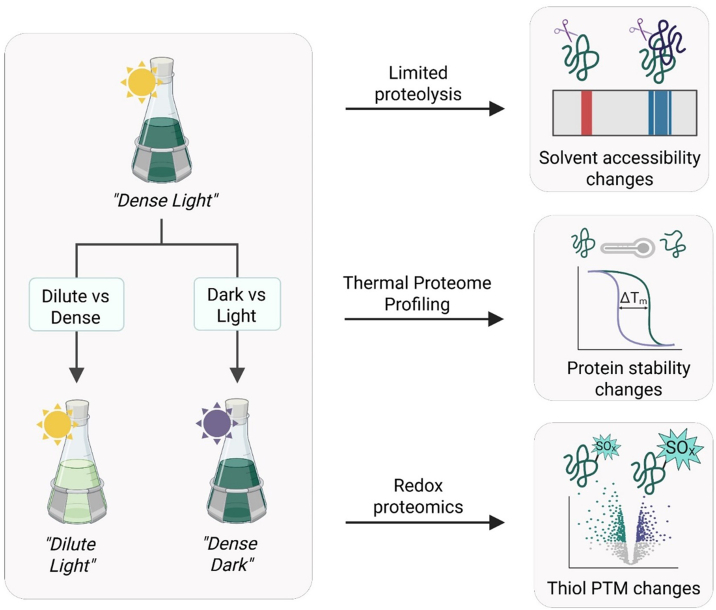
**Experimental design to investigate environmental adaptation responses of *Synechococcus elongatus*.** We grew cultures of *S. Elongatus* under constant light to an *A*_750_ of 1.0 which were defined as “dense”. To mimic a reduction in self-shading, we diluted *S. Elongatus* under light for 30 min (dense *versus* dilute). To study the acute impact of light disruption, we placed dense cultures in the dark for 2 h (*dark versus light*). We analyzed structural changes in the proteome in response to all perturbations using limited proteolysis, thermal proteome profiling, and redox proteomics. PTM, post-translational modification.

For structural proteomics studies, we introduced two perturbations to *S.* e*longatus* (Fig. 1). For both perturbations, we grew *S. elongatus* cultures under continuous light from *A*_750_ ∼0.15 to *A*_750_ ∼1.0 (late mid-exponential phase) over 4 days. After reaching desired absorbance, the culture was split into individual culture flasks that served as experimental replicates. We achieved the dilute disruption by diluting cultures in the experimental flasks from an *A*_750_ of 1.0 to *A*_750_ of 0.08 under constant light using filter-sterilized spent medium from the same cell culture. We maintained the dilute culture under light for 0.5 h until harvesting. We achieved light disruption by wrapping flasks with aluminum foil and placing in the dark for 2 h until harvesting. Nonperturbed cultures maintained at constant light and density served as controls. We harvested cultures by centrifugation (4700*g*, 5 min, 4 °C) and removal of the supernatant. We snap-froze cell pellets in liquid nitrogen and stored them at −80 °C until ready for lysis.

### Cell Lysis

We performed all proteomics studies on lysed *S. elongatus.* To lyse *S. elongatus* for LiP-MS and TPP-MS analysis, we resuspended frozen cell pellets from 10 ml of culture in 300 μl of filtered (0.45 μm) phosphate-buffered saline (PBS) (NaCl 137 mM, KCl 2.7 mM, Na_2_HPO_4_ 10 mM, and KH_2_PO_4_ 1.8 mM) at 4 °C and subjected pellets to bead-beating using a Bead Ruptor Elite (OMNI). We transferred the *S. elongatus* extracts to 0.5 ml polypropylene tubes (OMNI, Cat No. 19–623) containing 0.15 mm garnet beads and subjected samples to eight cycles of beating at 6.2 m/s. Each cycle consisted of 30 s of beating followed by 30 s in an ice bath to minimize sample heating. We centrifuged extracts (10,000*g*, 4 °C, 10 min), collected supernatant, aliquoted across tubes, and snap froze the samples, which we stored at −80 °C until use. When ready for use, we thawed protein extracts, centrifuged samples to remove debris and aggregates (10,000*g*, 4 °C, 10 min), collected the supernatant, and determined protein concentration using a bicinchoninic acid assay (BCA) assay (Thermo Fisher; Cat No. 23225).

### Proteomics Sample Preparation

#### Global *Proteomics*

We prepared samples for global proteomics analysis simultaneously with the LiP-MS. Briefly, we split *S. elongatus* extracts into two aliquots to undergo global proteomics analysis and LiP-MS analysis, which allowed us to tightly control for changes in protein abundance for the LiP-MS analysis (See the “*LiP-MS*”section for further details). For the global samples, we denatured proteins using 8 M urea and reduced the denatured proteins with 5 mM dithiothreitol for 30 min at 25 °C with constant shaking. We digested samples by adding sequencing-grade trypsin (Promega, Cat No. V5113) at an enzyme:substrate ratio of 1:100 with constant shaking at 850 rpm for 3 h. We quenched the digestion by acidifying the solution to 1% formic acid, cleaned the peptides using C18 solid-phase extraction, and quantified peptides for subsequent LC-MS/MS analysis with a BCA assay.

#### *LiP-*MS

We first treated the LiP sample with pronase, a nonspecific protease, at an enzyme: substrate ratio of 1:200. An equivalent volume of water was added to the global proteomics samples above as a control. We incubated all samples at 25 °C for 1 min, followed by a 5-min incubation at 98 °C, and denaturation with 8M urea. Post-denaturation, we digested samples and prepared for subsequent LC-MS/MS analysis as described above for the global proteomics samples.

#### *TPP-*MS

We diluted *S. elongatus* extracts to 1 mg/ml in PBS and aliquoted 150 μg into PCR tubes for temperature treatment. We treated samples across 10 temperatures from 37 to 82 °C in 5 °C increments, which we determined through optimization experiments (Supplemental Fig. S1). We incubated samples for 3 min at each respective temperature followed by 25 °C for 3 min on a ProFlex 3 x 32-well PCR System. In between steps, the samples were kept in ice batch to minimize unwanted temperature fluctuations. We pelleted the insoluble protein using centrifugation (18,200*g*, 4 °C, 66 min) and carefully removed 100 μl of supernatant for proteomic analysis. We took the supernatants to dryness, resuspended in 23 μl of 5% sodium dodecyl sulfate in 50 mM triethylammonium bicarbonate buffer (pH 8.5), and digested the protein using a S-Trap micro spin column system (Protifi; <100 ug) according to the manufacturer’s instructions. We digested peptides using sequencing grade trypsin at an enzyme:substrate ratio of 1:10 (μg:μg) overnight at 37 °C and eluted the peptides the following day according to the manufacturer’s instructions. We cleaned the peptides using C18 SPE and quantified peptide concentration using a BCA assay. We took the peptides to dryness, resuspended them in 500 mM HEPES buffer, and labeled peptides using a TMT10plex isobaric label reagent kit (ThermoFisher, Cat No. 90406) as previously described (29). The labeling scheme can be found in Supplemental Table S1. After combining labeled samples, we cleaned the peptides using C18 SPE and fractionated each TMT sample set into four fractions for subsequent LC-MS/MS analysis.

#### Redox*-MS*

We processed *S. elongatus* extracts as previously described (30). In contrast to structural proteomic workflows, where we need to maintain native structure integrity, harsher conditions can be used for lysing the cells for the redox proteomics workflow. Briefly, we resuspended and incubated *S. elongatus* cell pellets with 10% trichloroacetic acid on ice for 20 min. We then added 250 μl of lysis buffer (100 mM N-ethylmaleimide, 250 mM (4-(2-hydroxyethyl)-1-piperazineethanesulfonic acid, 10 mM ethylenediaminetetraacetic acid, 0.5% (w/v) sodium dodecyl sulfate, 8 M urea, pH 7.5) followed by incubation in dark with brief intermittent sonication for 2 h at 37 °C and beat beating. We collected lysate, precipitated proteins with 4× volume of cold acetone overnight at −20 °C, and digested the resulting protein samples with sequencing-grade trypsin. We used a TMT16plex isobaric label reagent kit (ThermoFisher, Cat No. A44520) to label peptides as previously described (29). The labeling scheme can be found in Supplemental Table S1. After combining the labeled samples, we cleaned the peptides and enriched cysteine (Cys)-containing peptides for subsequent LC-MS/MS analysis using resin-assisted capture (31).

### LC-MS/MS Data Acquisition

We performed LC-MS/MS analysis on peptides (typically 500 ng) from global, LiP-MS, and TPP-MS using a Thermo Scientific Q-Exactive HF-X mass spectrometer attached to a Thermo Dionex Ultimate 3000 chromatography platform. We configured the chromatography platform with two separate pumps for sample trapping and peptide elution. After sample trapping, we introduced and analyzed peptides over 60 minutes using a reverse phase C18 column (30 cm × 75 μm i.d., 1.7 μm particle size of Waters Acquity BEH particles, Waters) at 200 nl/min with buffer A (0.1% formic acid in H2O) and buffer B (0.1% formic acid in acetonitrile). We used the following gradient profile for peptide separation (min, %B): 0.0, 1.0; 2.6, 1.0; 6.3, 8.0; 53.5, 25.0; 58.5, 35.0; 61.3, 75.0; 62.8, 95.0; 65.5, 95.0; 66.0, 50.0; 67.0, 50.0; 67.6, 95.0; 71.0, 95.0; 71.3, 1.0; and 92.0, 1.0. We maintained the ion transfer tube temperature at 300 °C and the nanoelectrospray voltage at 2.2 kV. We acquired FT-MS spectra from 380 to 1800 m/z at a resolution of 60 k (AGC target 3e6) and selected the top 12 FT-HCD-MS/MS spectra in data-dependent mode using an isolation window of 0.7 m/z at a resolution of 45 k (AGC target 1e5). For HCD fragmentation, we used a normalized collision energy of 30 with a 45 s exclusion time, analyzing only charge states from 2 to 6. The data from the global proteomics samples were processed along with the LiP-MS samples (See the *limited proteolysis section* for further details). We analyzed redox proteomic samples using a Vanquish Neo UHPLC System(Waters) coupled to an Orbitrap Exploris 480 Mass Spectrometer [Thermo Scientific as recently described (31)].

### Data Processing

#### Global and LiP-MS Analysis

We searched the raw files (.raw) against a *S. elongatus* (strain ATCC 33912/PCC 7942/FACHB-805) UniProt database (Proteome ID #UP000889800, 2657 entries) using FragPipe 21.1 equipped with MSFragger 4.0 for label-free quantification. We processed the global proteomics sample (digested with trypsin alone) and LiP-MS sample (digested with both pronase and trypsin) separately in FragPipe with digestion mode selected as “semi-specific” for both searches. The precursor mass tolerance was set to ±20 ppm, fragment mass tolerance to 20 ppm, and allowed missed cleavages to 4. Searches also included N-terminal peptide acetylation and oxidation at methionine as variable modifications, an FDR of 0.01 at both the protein and peptide level, and match between run-match time window of 1.5 min. A summary of the database searching results for the global and LiP-MS datasets, including the sequence coverage and unique proteins identified for each protein is presented in Supplemental Table S2.

We analyzed output tables from FragPipe using the in-house python scripts available at (GitHub - PNNL-Predictive-Phenomics/Microbial_Isolate_LiP_Analysis: LiP analysis workflow of microbial isolates) to obtain protein- and peptide-level identifications and quantifications. We removed all contamination peptides and performed median center normalization across samples at both the protein-level and peptide-levels of quantification. For the global samples, we calculated log2-fold changes between control and each experimental group using student *t*-test *p*-values to show statistical significance levels of abundance changes. For those proteins with statistically significant changes in abundance, we normalized peptide values from the double-digested samples to account for underlying protein abundance differences and ensure changes in semi-tryptic peptides were due to conformational changes alone, not abundance changes. After normalization, we calculated log2-fold changes in semi-tryptic peptide abundance and performed a Student's *t*-test to determine statistical significance. We labeled each peptide as tryptic or semi-tryptic based on the digestion pattern. We further classified structurally altered proteins (*i.e.*, proteins with at least one statistically significant semi-tryptic peptide; *p* < 0.05) from the double-digested sample using the following parameters:

$$\text{Solvent}\;\text{Accessibility Score}=\frac{\text{Number}\;\text{of}\;\text{semi}\;\text{tryptic}\;\text{peptides}\;\text{in}\;\text{the}\;\text{double}\;\text{digested}\;\text{samples}}{\text{Number}\;\text{of}\;\text{fully}\;\text{tryptic}\;\text{peptides}\;\text{in}\;\text{the}\;\text{trypsin}\;\text{only}\;\text{samples}}$$

PerturbationScore=(SumofabsoluteFoldchangeofsignificantsemitrypticpeptides)×(SolventAccessibilityScore)

We classified proteins with perturbation scores less than one at level 1, proteins with perturbation scores between 1 and 4 at level 2, and proteins with perturbation scores greater than 4 at level 3. After processing, a small number of proteins that were only identified with a single peptide were preserved. We have added the annotated MS/MS spectra for these peptides in Supplemental Fig. S2.

#### Optimization of Nonspecific Protease in LiP-MS

We searched the raw files (.raw) generated by the Thermo mass spectrophotometer against a *S. elongatus* PCC7942 protein database (Release 2016, 2854 entries) using MaxQuant (Ver. 1.6.17.0) for label-free quantification. Each group of samples digested with the different proteases was processed separately, but with the same database searching parameters. The following settings were common to all the samples: (1) Digestion mode—“Semi-specific”, (2) variable modifications—N-terminal peptide acetylation, oxidation at methionine, (3) FDR at protein level—0.01, (4) FDR at peptide level— 0.01, (5) match between run-match time window—0.7 min, (6) minimum number of unique peptides that a protein group must have to be the final quantification table was set to 2, and (7) precursor mass tolerance was set to 20 ppm. The semi-specific search in MaxQuant does not allow the setting of the number of allowable missed cleavages. All other parameters were set to the default MaxQuant settings.

#### TPP Analysis

We searched raw MS data using MS-GF+ (release v2024.03.26) against *S. elongatus* PCC7942 protein database from Uniprot (Release 2016, 2854 entries) with the following parameters: a parent ion mass tolerance of 20 ppm, a partial tryptic rule of up to two missed cleavages, oxidation on methionine (+15.9949) as a variable modification, and TMT labeling on peptide N-termini and lysine residues (+229.1629) as a fixed modification. The precursor mass tolerance was set to 20 ppm. We extracted reporter ion intensities from TMT10 tags using MASIC (32) and used the R package PlexedPiper to link the MSGFPlus peptide identification and MASIC quantification results using an FDR of <1% at both the peptide and protein levels.

For each TMT dataset, we determined relative protein abundance by dividing the raw TMT intensities against the intensity at the lowest temperature. We used the relative protein abundance for data normalization among all 20 of the TMT10 plexes, using a procedure described in the TPP package (33) with modifications (Supplemental Table S1). Proteins for normalization (normP) had to be quantified in all TMT sets, have a relative abundance of the seventh highest temperature between 0.5 and 0.7, and a relative abundance of the 10th highest temperature point below 0.4. We used proteins in the TMT plex with the largest number of proteins that fulfilled the criteria as the normP. For each TMT set, we calculated the median relative abundance of normP and used it to fit a sigmoidal melting curve. We used the TMT set with the highest R^2^ as a reference for calculating a scaling factor to normalize all remaining TMT sets. After data normalization, curve fitting was performed for each individual protein. At least seven valid data points (out of a total of 10 in this experiment) were required for curve fitting. The T_m_ value for each protein was derived from the fitted model. To define proteins with significant change in T_m_, the student *t*-test was performed using the derived T_m_ values between two experimental groups.

#### Redox Analysis

We searched raw MS data using MS-GF+ (release v2024.03.26) against *S. elongatus* PCC7942 protein database from Uniprot (Release 2016, 2854 entries) and used MASIC to extract the TMT reporter ion intensity. The number of allowed missed cleavages was set to 2, and the precursor mass tolerance was set to 20 ppm. For multiplexed quantification, TMT labels on peptide N termini and lysine residues (+304.207146) were selected as fixed modifications. Oxidation on methionine (+15.9949), N-ethylmaleimide blocking of Cys (+125.047679), and alkylation of Cys (+57.0215) were selected as dynamic modifications. We filtered peptide spectra matches to only those with a mass accuracy <10 ppm and PepQ value < 0.01. We log2-transformed the TMT reporter ion intensity data and aggregated to unique Cys sites. Statistical comparison was performed using the student *t* test.

### Functional Enrichment Analysis

We performed a functional enrichment analysis using the R package clusterProfiler 4.0 (34) using gene annotations from the Gene Ontology (GO) (35) and Kyoto Encyclopedia of Genes and Genome (KEGG) databases (36) curated for cyanobacterial proteins (37). We determined *p*-values for enriched terms for multiple testing using the Benjamini-Hochberg correction and included only enriched terms with corrected *p*-values less than 0.05. For global proteomics and TPP-MS datasets, we included any proteins with a student’s *t*-test *p*-value less than 0.05. For LiP-MS, we performed enrichment on proteins at each structural perturbation level. For redox proteomics, we included proteins with unique oxidation sites with a *p*-value less than 0.05 that we mapped to a nonredundant list of proteins.

### Experimental Design and Statistical Rationale

The experimental design has been described in detail under the cyanobacterial sample harvest methods section. In brief, samples grown under constant light conditions were used as controls, while the experimental perturbations involved either a 30-min light intensification or a 2-h light disruption. Each sample was split across the three techniques and centrifuged to obtain the cell pellets. Five experimental replicates were used for LiP and TPP-MS, while three replicates were used for redox proteomics. The data acquisition, database searching, and analysis parameters for each individual method are described in detail above. The TMT plex designs for multiplexed quantification experiments are presented in Supplemental Table S1. We applied student t-tests on log2 intensities to determine statistical significance. False discovery rates were estimated using the target decoy approach. The results from the over-representation enrichment analysis were filtered using adjusted *p*-value cut-off of 0.05. All other relevant parameter files, outputs from database searches, and associated metadata are available in the MassIVE repository.

## Results

### Study Design Mimicking Physiologically Relevant Light Conditions

Our goal was to deeply probe the molecular responses of the model cyanobacterium *S. elongatus* to biologically relevant environmental perturbations that directly impact photosynthetic efficiency and metabolic regulation in both natural ecosystems and industrial bioproduction systems. Cyanobacteria experience simultaneous variations in cell density and light availability. In natural environments, water column mixing, cloud cover, and diurnal cycles create dynamic transitions between light-limited and light-saturated conditions, while population growth during bloom formation progressively intensifies self-shading that further limits light penetration. Similarly, in industrial photobioreactors, achieving economically viable biomass concentrations inevitably creates self-shading effects where exterior cells experience light saturation while interior cells face light limitation. Industrial cultivation involves stepwise scale-up from laboratory flasks to large-scale production systems, with each transfer of a smaller culture volume into a larger vessel creating dilution events that produce abrupt density-driven changes in light exposure. These density-dependent light gradients represent a fundamental challenge in both natural bloom dynamics and industrial cultivation optimization.

The goal of our study was to mimic these real-world conditions to investigate cyanobacteria responses to these environmental shifts. To do so, we used a systems biology approach where we coupled traditional proteomics approaches that quantify protein abundance changes with structural proteomics tools that capture dynamic conformational adaptations of the proteome that enable the cyanobacteria to adapt to these environmental shifts. We subjected *S. elongatus* to two physiologically meaningful perturbations: (1) dilution under constant light and (2) transient light disruption (Fig. 1). The dilution perturbation simulates the transition from intermittent light availability due to self-shading in dense cultures (*A*_750_ = 1.0) to constant exposure to increased light in diluted cultures (*A*_750_ = 0.08). We maintained cultures under the same continuous light for 30 min after dilution to capture the rapid structural adaptations that enable cyanobacteria to optimize their cellular machinery for enhanced light exposure under a “no-shading” condition. The dark treatment represents an acute light limitation perturbation, such as regular day-night transitions or unexpected weather interruptions, that require immediate metabolic adjustments. We placed dense cultures (*A*_750_ = 1.0) grown under continuous light into darkness for 2 h before harvesting. The starting dense cultures maintained under constant light served as our controls for both perturbations. Following each treatment, we harvested cells and applied four complementary proteomic approaches—global proteomics, LiP-MS, TPP-MS, and redox proteomics—to comprehensively characterize both abundance and structural changes in the proteome response.

### Global Proteomics

**Fig. 2:**
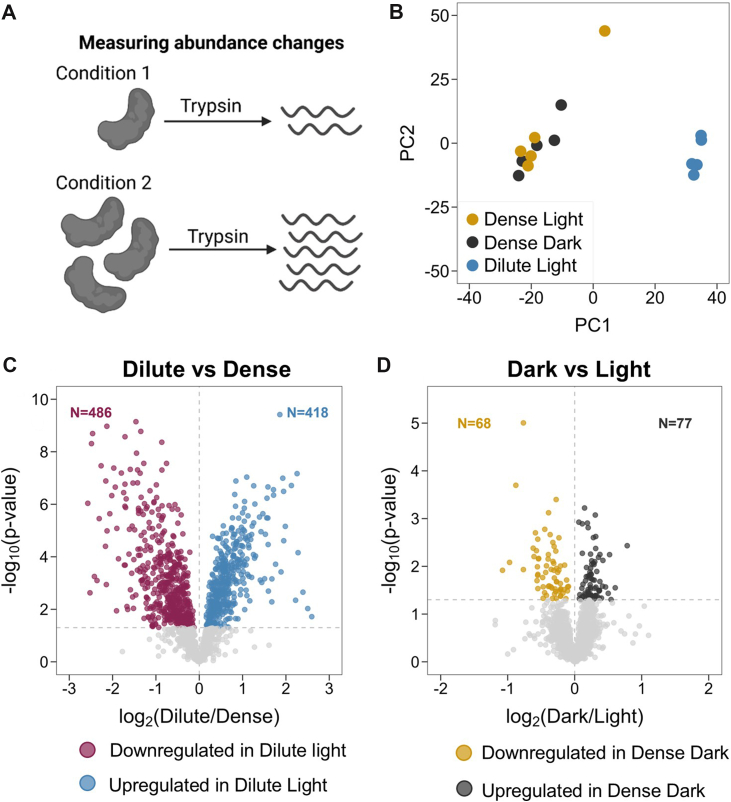
**Impact of environmental perturbations on protein expression in *S. elongatus*.***A*, protein extracts from *S. elongatus* before and after environmental perturbations are denatured and digested with trypsin. Changes in the abundance of tryptic peptides are subsequently analyzed using LC-MS/MS. *B*, a principal component analysis of identified proteins with altered expression across different experimental groups. *C* and *D*, volcano plots from individual changes in protein expression after (*C*) dilution and (*D*) removal of light.

First, we performed traditional global proteomics to measure differential protein expression in *S. elongatus* after each perturbation. We subjected protein extracts obtained from *S. elongatus* before and after each environmental perturbation to denaturation and proteolytic digestion with trypsin (Fig. 2*A*). Trypsin specifically cleaves the C-terminal side of lysine and arginine residues of the amino acid backbone of the proteins to create tryptic peptides that we analyzed by LC-MS/MS. We identified 1897 total proteins across all our experimental groups (Supplemental Table S3). A principal component analysis (PCA) of protein abundance patterns revealed the magnitude of cellular responses triggered by applied light perturbations (Fig. 2*B*). Diluted samples exposed to increased light availability exhibited clear separation from dense control samples in their PCA distribution, indicating comprehensive proteomic changes as cells transitioned from intermittent light exposure to continuous light exposure in diluted cultures. In stark contrast, cultures placed in the dark for 2 hours showed minimal separation from dense controls, suggesting that transient darkness did not sufficiently alter intracellular conditions to trigger major global proteome changes.

These observations are reflected in the volcano plots that depict changes in protein abundance at the individual level following dilution (Fig. 2*C*) and dark treatment (Fig. 2*D*). Dilution had a pronounced impact on protein abundance, with changes in abundance of over 900 different proteins, indicating rapid and extensive proteome reorganization as cells respond to unobstructed light penetration. Among these changes, 418 proteins showed increased abundance, while 486 proteins displayed decreased abundance. In comparison, dense cultures exhibited a modest change in 145 proteins, with 77 proteins showing increased abundance and 68 proteins displaying decreased abundance. These observations suggest that transient darkness triggers limited proteomic adjustments which are potentially focused on maintaining essential cellular functions during an unexpected period of light deprivation.

### Measuring Changes in Solvent Accessibility With LiP-MS

**Fig. 3:**
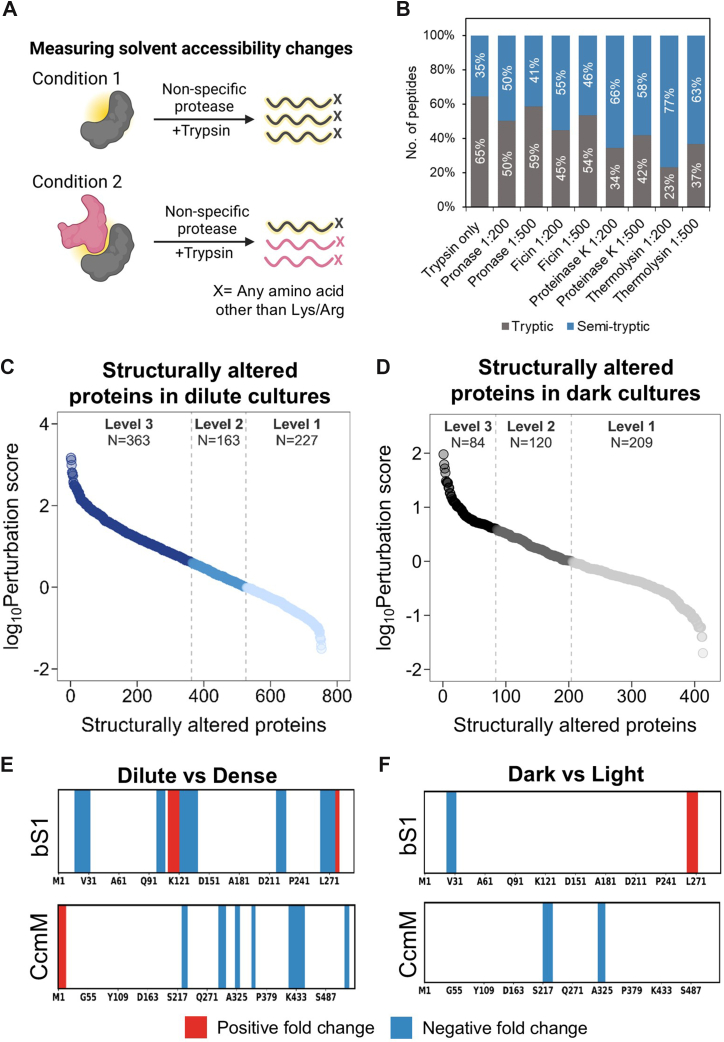
**Identifying the effect of environmental perturbations on *S. elongatus* protein solvent accessibility using LiP-MS.***A*, protein extracts were briefly digested with a nonspecific protease, then denatured and digested with trypsin. This double digestion produces semi-tryptic peptides (cleaved at lysine/arginine on one end and any other amino acid on the opposite end), which report on solvent accessibility changes. *B*, after testing several nonspecific proteases and ratios, the digestion conditions were optimized to pronase (enzyme:substrate = 1:200). *C* and *D*, scatter plots show the classification of proteins with altered solvent accessibility at different levels following (*C*) dilution and (*D*) dark treatment. *E* and *F*, the structural barcodes for selected proteins are shown for (*E*) dilute *versus* dense and (*F*) dark *versus* light comparisons. The colored regions indicate semi-tryptic peptide sequences that show significant differences in solvent accessibility. LiP-MS, limited proteolysis-mass spectrometry; bS1, small ribosomal subunit protein bS1 (Synpcc7942_1591); CcmM, carboxysome assembly protein (Synpcc7942_1423).

To complement the global proteomics data, we employed LiP-MS to probe proteome-wide structural changes in response to the environmental perturbations. LiP-MS leverages the activity of nonspecific proteases, which selectively digest solvent-accessible regions of proteins, providing insights into protein conformational shifts across experimental conditions (Fig. 3*A*). Unlike trypsin, which selectively cleaves at lysine and arginine residues, nonspecific proteases are agnostic to protein sequence and thus cleave accessible regions across the protein surface. After reaction and rapid quenching *via* denaturation, we subject the partially digested proteins to trypsin to produce both fully tryptic and semi-tryptic peptides, which we analyzed by LC-MS/MS. Quantifying changes in the semi-tryptic peptide pattern enables identification of proteins undergoing structural remodeling, connecting solvent accessibility dynamics to functional adaptations in response to light conditions. Crucially, we normalized all LiP-MS data to protein abundance changes (as measured by global proteomics above) to ensure observed structural changes were solely attributable to altered conformations rather than protein upregulation or downregulation.

To ensure robust and reproducible detection of solvent-accessible regions without over-digesting protein samples, we first optimized the protocol by screening multiple nonspecific proteases—pronase, ficin, proteinase K, and thermolysin—at two enzyme-to-substrate ratios (1:200 and 1:500). We included a trypsin-only digestion control to account for the presence of endogenous protease activity, which naturally generates semi-tryptic peptides even in undigested samples (Fig. 3*B*). For the trypsin-only control, semi-tryptic peptides comprised ∼35% of the total peptides detected, indicative of native protease activity within the system. From our tests, we determined that pronase at an enzyme-to-substrate ratio of 1:200 to be optimal for digestion. Pronase digestion resulted in a balanced peptide population (∼50% semi-tryptic peptides, 50% fully tryptic peptides), ensuring reliable detection of conformational changes while maximizing the yield of semi-tryptic peptides relative to the trypsin-only digestion control. This balance was critical for accurately capturing structural dynamics while minimizing protease activity on protein cores.

To quantify the relative impact of structural change on a protein in response to the two perturbations, we implemented two metrics: solvent accessibility score and perturbation score (*see Methods for details)*. The solvent accessibility score quantifies the number of semi-tryptic peptides generated from surface regions of a given protein, normalized by protein size. In essence, it reflects the degree of folding or unfolding of a protein. Perturbation score builds on this by integrating the fold changes in abundance of a statistically significant semi-tryptic peptides between two conditions, providing a quantifiable measure of structural impact. We validated both methods to ensure no bias toward size of a protein (Supplemental Fig. S3). Using these metrics, we classified structurally altered proteins into three tiers: level 1: minimal structural changes (perturbation score <1), level 2: moderate structural changes (perturbation score 1–4), and level 3: prominent structural changes (perturbation score >4). This classification framework allowed us to better identify cellular processes and macromolecular complexes linked to environmental perturbations opposed to natural dynamics in the system.

In response to increased light availability from dilution, we observed 753 proteins showing structural alterations (Supplemental Table S4). Applying predefined perturbation score thresholds, 227 proteins were classified as level 1, 163 proteins as level 2, and 363 proteins as level 3 (Fig. 3C). These results indicate widespread structural changes that reflect cyanobacterial adaptation to optimize cellular machinery for enhanced light availability when self-shading is eliminated in dilute cultures. Short-term exposure to darkness induced structural alterations in 413 proteins, with 209 proteins classified as level 1, 120 proteins as level 2, and only 84 proteins as level 3 (Fig. 3D and Supplemental Table S4). Compared to the increase in light exposure due to dilution, the reduction in light exposure elicited fewer structural changes, suggesting that transient light deprivation (2h dark treatment) exerts a more subtle impact on cyanobacterial protein conformations than the shorter exposure to increased light availability (30 min light exposure *via* dilution). However, despite the smaller magnitude of structural changes compared to light-rich environments, we detected nearly 3-fold more proteins with structural alterations under light/dark conditions (413 proteins) than with changes in abundance alone (145 proteins) in the same samples. This divergence underscores the sensitivity of LiP-MS in detecting structural responses to mild perturbations, revealing conformational adaptations that occur without changes in protein abundance.

The LiP-MS workflow further enables the identification of specific protein regions with altered solvent accessibility, visualized using 2D barcodes—heatmaps that depict significant semi-tryptic peptide fold changes mapped along the amino acid sequence of individual proteins. To showcase the informational depth of LiP-MS and clarify its visualization strategy, we highlight two barcodes as illustrative examples. Barcodes for two representative proteins, small ribosomal protein bS1 (coded by *rpsA2*) and carboxysome assembly protein CcmM, illustrate structural changes observed after an increase in light exposure (Fig. 3*E*) or complete removal of light (Fig. 3*F*). The bS1 protein is a subunit of the bacterial ribosome’s small subunit and is a critical regulator of translation through binding of mRNA. CcmM is a scaffold protein that plays a key role in the concentration of the enzyme RuBisCO which is responsible for carbon fixation (38). Regions of increased solvent accessibility correspond to semi-tryptic peptides with positive fold changes in the treatment group relative to controls and are visualized as shades of red in the barcodes. Thus, after increased exposure to light, the bS1 peptide spanning residues 110 to 121 and the CcmM peptide spanning residues 2 to 14 have increased solvent accessibility, indicating more open conformations that may allow functional adjustments in response to enhanced light conditions. Conversely, regions shown in shades of blue represent peptides with negative fold changes, signifying reduced solvent accessibility. For example, after increased exposure to light, the semi-tryptic peptide of bS1 spanning residues 17 to 32 exhibited decreased accessibility, suggesting this region became less exposed on the protein surface due to a potential increase packing in this region or perhaps an interaction with a partner protein or metabolite that masks the region from the protease. Overall, we observed more structural changes in both bs1 and CcmM after increased exposure to light compared to shifting the cultures to the dark, consistent with the notion that greater light exposure results in a more robust response from *S. elongatus*. Collectively, these results provide detailed insights into how individual protein regions undergo dynamic conformational and potentially functional changes in response to environmental perturbations that were not evident from abundance-based approaches alone.

### Measuring Thermal Stability Shifts With TPP-MS

While LiP-MS provides detailed insights into structural rearrangements within solvent-accessible regions of proteins, it does not reveal what initiates these events or whether they result in stabilization or destabilization of the protein. TPP fills this gap by quantifying shifts in the thermal stability of proteins, which primarily arise from associations or dissociations with protein interaction partners, metabolites, or ligands along a given regulatory pathway (33). As association and dissociation of binding events increase and decrease a protein’s thermal stability respectively, TPP complements LiP-MS by providing directionality to the structural changes and the potential to link structural changes to molecular interactions. We reasoned that together, these methods would enable a more comprehensive understanding of proteome-wide structure-function dynamics, bridging insights from conformational changes to system-level interactions.

TPP works by leveraging a thermal gradient applied to protein lysates to assess the temperature-dependent stability and solubility of proteins. Upon reaching their intrinsic melting temperature (T_m_), proteins unfold and become insoluble and aggregate. These insoluble protein aggregates can then be separated from the soluble proteins using high-speed centrifugation (23) leaving only the soluble fraction which contains proteins that remain stable at each temperature point. These proteins are subsequently collected and processed for quantitative proteomics analysis. By using isotope-coded isobaric mass tags, peptides processed from the soluble proteins at each temperature can be individually labeled, allowing recombination of the samples along the temperature gradient for a single run on the LC-MS/MS. This ensures high-precision measurements of changes in protein solubility across the temperature gradient and the ability to generate high-quality melting curves for each individual protein by fitting to the reporter ion intensities. The specific T_m_ of a given protein can then be determined from the inflection point within the melting curve. By determining the perturbation-specific changes in the T_m_ of a protein, we can identify how the perturbation impacts protein stability, and consequently, potential binding dynamics.

**Fig. 4:**
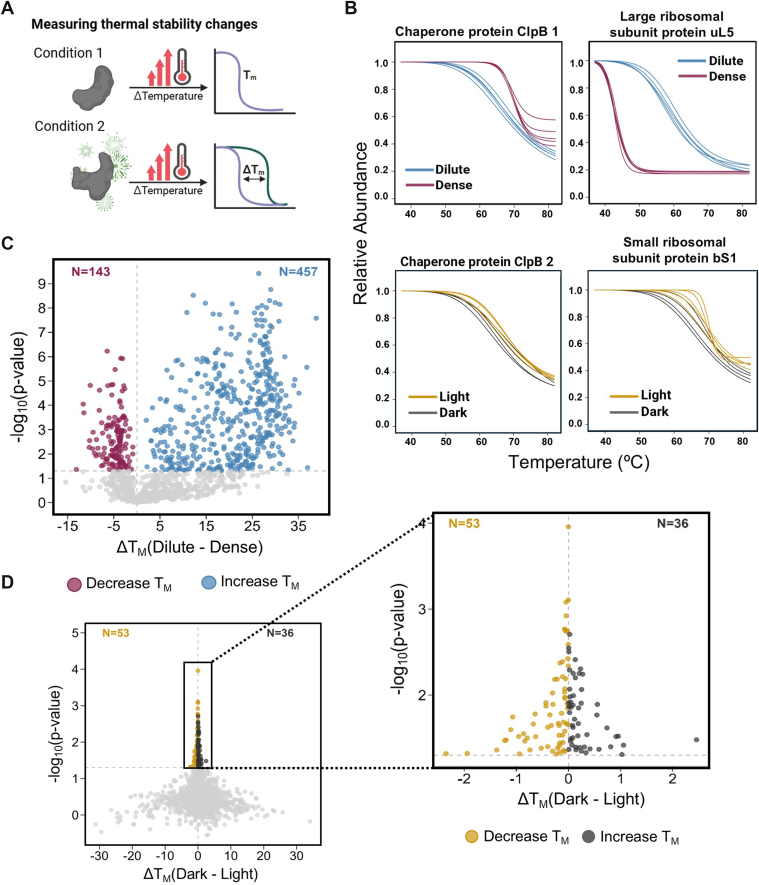
**Identifying proteome-wide thermal stability changes by capturing melting temperatures with TPP.***A*, a scheme representing the TPP basic principle is shown. Samples are heated across a temperature gradient to generate proteome-wide melting curves and identify thermal stability changes. *B*, representative melting curves for selected proteins comparing the dense to dilution condition (*top*) and *light to dark* conditions (*bottom*). *C* and *D*, volcano plots showing the differential protein expression caused by (*C*) dark treatment and (*D*) reduction in cell shading induced by dilution. TPP, thermal proteome profiling.

Initially, we optimized our TPP protocol by applying a broad temperature gradient to protein extracts of *S. elongatus* and quantifying changes in the total protein abundance in the soluble fraction at each temperature. Our global protein melting curve (Supplemental Fig. S1) indicated that the *S. elongatus* proteome begins to destabilize around 50 °C and completely unravels around 80 °C. Based on these studies, we further refined the temperature gradient to use 10 temperatures in five-degree increments spanning the range of 37 to 82 °C (Fig. 4*A*, Supplemental Fig. S1, *see* Experimental Procedures), allowing us to employ a TMT10 labeling kit for each temperature for the generation of downstream melting curves.

We next applied our technique to the protein extracts of *S. elongatus* to monitor changes in thermal stability across the two experimental conditions. In total, we identified 1581 proteins across the experimental groups (Supplemental Table S5). We then generated melting curves for all the detected proteins based on quantitative measures of protein abundance at each temperature (Supplemental Fig. S4, *see Material and Methods*). A key first measure of the quality of the data is reflected in the shape of the melting curves, where curves exhibiting a “proper shape” are more likely to represent a real unfolding event (33), demonstrating that the loss of protein is due to perturbation-induced changes in the melting temperature of the protein, not just changes in the aggregation properties of the protein. This is evidenced by the high quality of fit for our data where we observe a median R^2^ > 0.96 of the sigmoid curves in a majority of our datasets in-line with previous TPP reports (23). Using these thresholds, we generated 1231 curves for proteins after increased exposure to light and 1255 curves for proteins after removal of light and four representative curves are shown (Fig. 4*B*). We identified changes in the melting temperature of chaperone protein (ClpB), a molecular chaperone involved in stress response. We observed that the melting temperature of ClpB increased after increased light exposure and modestly decreased after removal of light, indicating increased activity and interaction after increased exposure to light. Additionally, we also observed several protein subunits of the ribosome to be impacted by the experimental conditions. After exposure to increased light, we observed a remarkable increase in the melting temperature of the large ribosomal subunit protein uL5, indicating a clear upregulation of protein synthesis after dilution. On the other hand, we observed a decrease in the small ribosomal subunit protein bS1 after removal of light, indicating protein synthesis is likely attenuated when placed in the dark. Notably, these proteins revealed excellent curve shapes and agreements between experimental replicates.

After fitting curves, we calculated changes in T_m_ for each protein before and after each perturbation. As observed with protein abundance changes and LiP-MS structural changes, exposure to increased light elicited a strong response with 600 proteins showing a shift in T_m_ (Fig. 4*C*). Remarkably, these changes in T_m_ ranged from −15 °C up to 35 °C with the majority, 457 proteins, experiencing an increase in T_M_ after dilution. This indicates a strong shift toward increased protein interactions with binding partners in response to increased light availability, likely driving more activated cellular machinery. On the other hand, removal of light resulted in only 89 proteins experiencing a significant shift in T_m_ (Fig. 4*D*) despite the generation of a larger number of total melting curves in this condition. The range of shift was also relatively minor (±3 °C) compared to increased exposure to light, indicating less significant change in protein interactions after light removal. These results provide an additional layer of molecular insight into how and to what degree each perturbation may be inducing structural changes.

### Measuring Changes in Cys Oxidation States With Redox Proteomics

Not all structural fluctuations observed in the proteome are induced by binding interactions; PTMs, such as oxidative modifications of Cys thiols, also play critical roles in driving structural changes and functional regulation. Redox modifications of Cys residues are central to cellular signaling and stress responses in a broad variety of organisms, including cyanobacteria (39, 40). Reactive Cys residues are particularly sensitive to fluctuations in the intracellular redox environment, such as increases in reactive oxygen species (ROS) and oxidative stress. These oxidative modifications act as molecular switches, enabling proteins to serve as "redox sensors" that regulate activity, structural integrity, and molecular interactions in response to environmental perturbations. Given the well-documented importance of redox regulation in photosynthesis (41) and metabolic adaptation (42, 43), we sought to quantify changes in Cys oxidation levels in *S. elongatus* induced by light fluctuations. This analysis thus complements our LiP-MS and TPP structural approaches by addressing critical PTM-mediated changes to the protein.

**Fig. 5:**
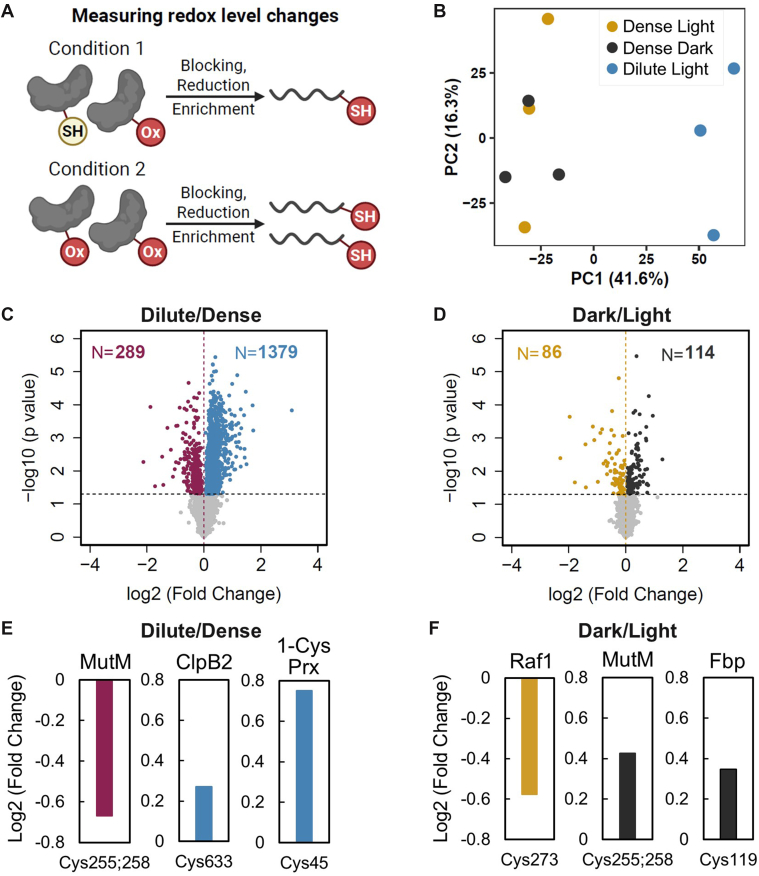
**Enrichment and analysis of cysteine (Cys)-containing peptides from the *S. elongatus* proteome after light perturbations.***A*, the workflow of redox proteomics. Protein free thiols are blocked *via* alkylation to prevent thiol-disulfide exchange and preserve the *in situ* redox proteome. Oxidized Cys thiols are reduced, and the Cys-containing peptides are enriched using a thiol-affinity resin for quantitative proteomics analysis. *B*, PCA plot showing the separation of different experimental conditions. *C*, Cys-containing peptide abundance changes after increased exposure to light. *D*, Cys-containing peptide abundance changes after removal of light. *E*, protein oxidation level change for selected Cys sites in MutM, ClpB2, and 1-Cys Prx after increasing exposure to light. *F*, protein oxidation level change for selected Cys sites in Raf1, MutM, and Fbp after increase exposure to light. Fbp, Fructose-1,6-bisphosphatase class 1; MutM, formamidopyrimidine-DNA glycosylase; ClpB2, chaperone protein ClpB 2; Raf1, RuBisCO accumulation factor 1.

Using a well-established workflow (44, 45, 46) (Fig. 5*A*), we preserved the endogenous redox state of the proteome by blocking unoxidized “free” thiols with an alkylation reagent to prevent dynamic thiol-disulfide exchange. We then digested proteins and reduced oxidized Cys residues on the peptides with dithiothreitol to expose the previously occupied thiol groups. This allowed us to enrich the reduced peptides through covalent binding to a thiol-affinity resin. We performed quantitative proteomics analysis using TMT-labeling and LC-MS/MS to provide insights into differential Cys oxidation levels across experimental conditions. Importantly, each TMT-set included controls prepared for global proteomics analysis to account for variations in protein abundance that might skew the interpretation of changes in oxidized peptides.

Our analysis identified 4716 unique Cys sites across 1600 proteins in *S. elongatus* proteomes under the different light exposure conditions (Supplemental Table S6). Redox proteomics revealed substantial oxidative modifications triggered by increased light exposure compared to transient light removal. PCA demonstrated clear separation between dilute cultures exposed to more light and dense cultures under constant light or transient dark conditions, with PC1 accounting for 41.6% of total variation (Fig. 5*B*). In contrast, transient dark conditions resulted in smaller shifts in Cys oxidation patterns, as evidenced by reduced PCA separation between these samples and dense cultures under constant light.

Increased light exposure drastically shifted the proteome toward a more oxidized state, resulting in modifications of 1668 Cys sites across 960 proteins. Among these, 1379 sites showed increased oxidation, while 289 sites exhibited decreased oxidation (Fig. 5*C*). In agreement with known oxidative stress responses, we observed increased oxidation levels in key ROS-regulating proteins such as superoxide dismutase, thioredoxin, 2-Cys peroxiredoxin, and catalase-peroxidase (Supplemental Table S6). These proteins play crucial roles in mitigating oxidative damage, which is expected to be exacerbated under enhanced light exposure. In comparison, transient darkness resulted in changes of only 200 Cys sites across 144 proteins, with 114 sites showing increased oxidation and 86 sites showing decreased oxidation (Fig. 5*D*). These more balanced shifts suggest stress modulation and energy conservation mechanisms, rather than the large-scale oxidative reprogramming, consistent with the minimal structural rearrangements observed in these samples *via* LiP-MS and TPP.

Next, we delved further to identify proteins involved in oxidative stress that contain Cys residues directly in their active sites, indicating they are specifically modulating stress response through redox. For example, MutM—a DNA glycosylase responsible for repairing oxidized purines in DNA (47)—showed decreased oxidation during light exposure (Fig. 5*E*) but increased oxidation after transient darkness (Fig. 5*F*). These inverse oxidation patterns align with expected fluctuations in oxidative stress between the two different perturbations. These data also suggest that most repair activity occurs after removal of light, where we see an increase in MutM oxidation state, suggesting increased binding to oxidized purines. Conversely, we observed increased oxidation of the molecular chaperone ClpB after increased light exposure. Coupled with the decreased thermal stability changes observed by TPP, these oxidation events may indicate disrupted interactions with binding partners and impaired chaperone function due to free radical damage. Notably, ClbB showed no changes in protein abundance, suggesting its functional regulation is driven entirely by redox PTMs at distinct Cys residues. Additional significant oxidation events found in proteins that modulate stress-response were observed in 1-Cys peroxiredoxin (Cys45) (Fig. 5*E*) after increased exposure to light and fructose-1,6-bisphosphatase (Cys119) after limiting light (Fig. 5*F*), the latter also being identified by LiP-MS. Collectively, these observations suggest that elevated ROS production and redox signaling activity from enhanced metabolic activity in the diluted cultures drive structural modifications and functions beyond what can be inferred from changes in abundance alone. Further, these underscore the complementary nature of the different structural measures, which can provide mechanistic insights into how these proteins modulate adaptive responses to environmental perturbations such as oxidative stress.

### Identifying the Altered Biological Processes With Functional Enrichment Analysis

**Fig. 6:**
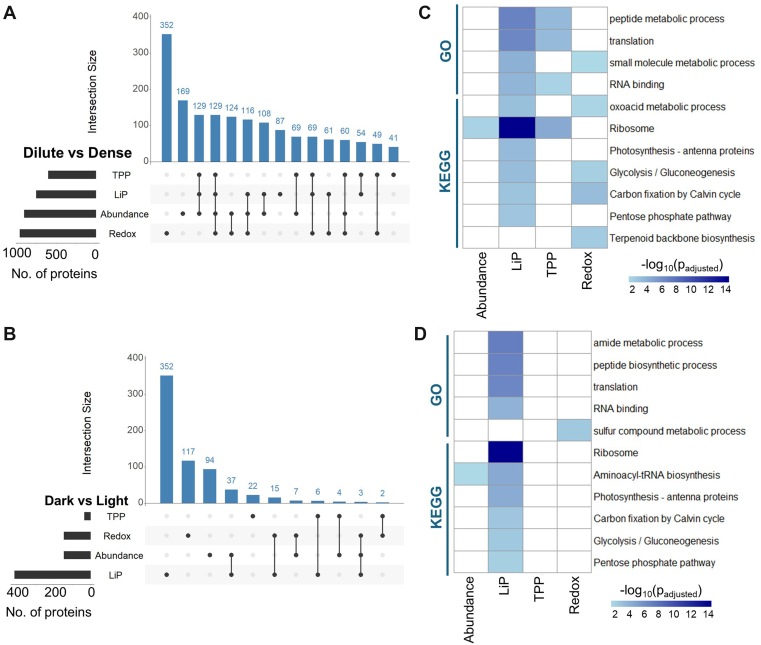
**Comparison of global, LiP-MS, TPP-MS, and redox proteomic datasets at protein and pathway levels.***A* and *B*, the overlap of significant proteins across the four techniques are visualized using Upset plots for (*A*) dilute *versus* dense comparison and (*B*) *dark versus* light comparison. *C* and *D*, the results of functional enrichment analyses performed to identify the GO terms and KEGG pathways associated with the significant proteins are presented as heatmaps for (*C*) dilute *versus* dense comparison and (*D*) light *versus* dark comparison. KEGG, Kyoto Encyclopedia of Genes and Genome; LiP-MS, limited proteolysis-mass spectrometry; TPP-MS, thermal proteome profiling-mass spectrometry; GO, Gene Ontology.

With a comprehensive readout of proteins undergoing abundance, structural, and redox changes, we next sought to leverage the data to identify key functional pathways altered in *S. elongatus* in response to the environmental perturbations. To evaluate the overlap in the proteins identified by each method, we generated UpSet plots comparing significantly altered proteins following increased light exposure (Fig. 6*A*) or transient light deprivation (Fig. 6*B*). Again, the level of proteomic alteration captured across the different approaches was remarkably higher in *S. elongatus* exposed to more light compared to changes in light deprivation (1618 *versus* 660 overall proteins). Among the complementary approaches, redox proteomics identified the largest number of unique protein changes after increased light exposure, mapping oxidative modifications to 352 proteins (Fig. 6*A*). Changes in protein abundance identified the next highest with 169 unique proteins, followed by LiP-MS with 87 proteins and TPP with 41 proteins. Remarkably, we found that only 129 proteins (<10%) were significantly altered across all four techniques after increased light exposure, emphasizing how each approach provides distinct and complementary molecular insights into cellular adaptation. In contrast, no proteins were identified across all four proteomics methods after transient dark treatment (Fig. 6*B*), and the largest number of unique changes was detected in the LiP-MS dataset. These findings suggest a heightened sensitivity of LiP-MS, which appears to capture structural responses that may remain undetected by other techniques under milder perturbations such as transient darkness.

To identify the biological pathways associated with the significant proteomic changes, we performed functional enrichment analysis using GO categories and KEGG databases. A comprehensive list of enriched GO terms and KEGG pathways is provided in Supplemental Table S7, while selected results are shown in Figure 6, *C* and *D*. Strikingly, despite global proteomics identifying the second-highest number of unique protein changes after increased light exposure, only translation-related processes such as ribosome and aminoacyl-tRNA biosynthesis, were significantly enriched using proteins within this dataset (Fig. 6*C*). Additional translation-associated pathways were enriched among significant proteins detected by LiP-MS and TPP-MS datasets, highlighting major alterations in ribosomal machinery captured by multiple proteomic approaches in response to enhanced light penetration. In contrast, redox proteomics revealed significant enrichment of metabolic pathways, including carbon metabolism and glycolysis, which were also captured by LiP-MS, suggesting coordinated regulation between protein structural dynamics and redox modifications. We also observed structural changes in the photosynthesis–antenna proteins pathway uniquely captured by LiP-MS with major remodeling of the C-phycocyanin beta and alpha subunits, as might be expected with increased exposure to light.

Despite much lower identifications of changes in the proteome, we observed similar trends in the functional enrichment analysis of proteins altered due to transient dark exposure (Fig. 6*B*). Pathways related to translation and protein translation machinery were exclusively enriched within the LiP-MS dataset, while pathways involving broader metabolism did not reach significance in TPP or redox proteomics datasets. To assess the contributions with different levels of structural perturbation, we segregated the proteins within the LiP-MS dataset into three different levels based on structural changes—minimal (Level 1), moderate (Level 2), and extensive (Level 3)—and repeated the enrichment analysis for each group (Supplemental fig. s5). The results revealed that enriched pathways under both perturbations were largely driven by level 3 proteins, indicating that proteins with the most pronounced structural changes play key roles in pathway regulation. Collectively, these enrichment approaches show how the complementary structural proteomics technologies provide a more detailed understanding of the enriched pathways and macromolecular complexes within the pathways that are potentially regulating the adaptive response of *S. elongatus*.

### Comparative Analysis of Metabolic Pathways and Enriched Complexes

**Fig. 7:**
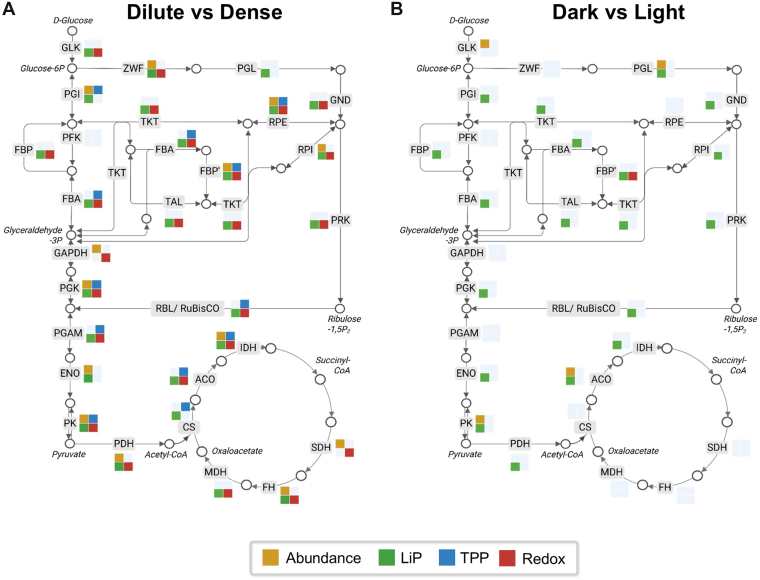
**Mapping the abundance and structural changes in proteins associated with carbon metabolism.** The metabolic network is adapted from the KEGG carbon metabolism pathway (syf01200). *A* and *B*, the proteins with statistically significant differences between the (*A*) dilute and dense samples, and the (*B*) *dark and light* samples are depicted with colored squares: global proteomics (*yellow*), LiP-MS (*green*), TPP-MS (*blue*), and redox proteomics (*red*). KEGG, Kyoto Encyclopedia of Genes and Genome; KEGG, Kyoto Encyclopedia of Genes and Genome; LiP-MS, limited proteolysis-mass spectrometry; TPP-MS, thermal proteome profiling-mass spectrometry.

Given the notable differences in enrichment across the proteomics platforms, we sought to more deeply investigate individual components of specific pathways to better understand the insights provided by each technology. To begin, we focused on the carbon metabolism pathway, as it is well characterized and showed convincing evidence of perturbation in LiP-MS datasets under both light exposures (Fig. 6). We reasoned that this pathway would serve as an excellent comparative example to explore how major and minor environmental perturbations impact cellular processes. Within the pathway, we examined individual protein components from glycolysis, the pentose phosphate pathway, and both the Calvin and TCA cycles (Fig. 7). The UniProt identifiers for these proteins are shown in Supplemental Fig. S6. Remarkably, exposure to increased light revealed changes in nearly every protein within the carbon metabolism pathway (Fig. 7*A*). We observed several proteins impacted by increased light exposure that were detected by changes in abundance and/or TPP despite a lack of enrichment in these pathways when analyzing the individual datasets. Key enzymes such as Fba and RuBisCo exhibited significant structural changes without corresponding changes in protein abundance, highlighting the complementary nature of the structural proteomics technologies and demonstrating their ability to fill in the gaps that traditional abundance-focused approaches often miss.

In contrast, transient removal of light resulted in fewer changes in components of the metabolic pathways (Fig. 7*B*). TPP datasets failed to detect changes in any of these proteins, while redox and global proteomics identified only a handful of affected proteins. Remarkably, LiP-MS alone detected structural alterations in 18 out of the 28 core proteins in the pathway, with 14 being at a level 2 or higher, highlighting its sensitivity in detecting more subtle responses to environmental perturbations. Taken together, these results suggest that while TPP and redox proteomics provide significant biological insights, LiP-MS may be uniquely suited for assessing adaptive responses under less pronounced perturbations.

**Fig. 8:**
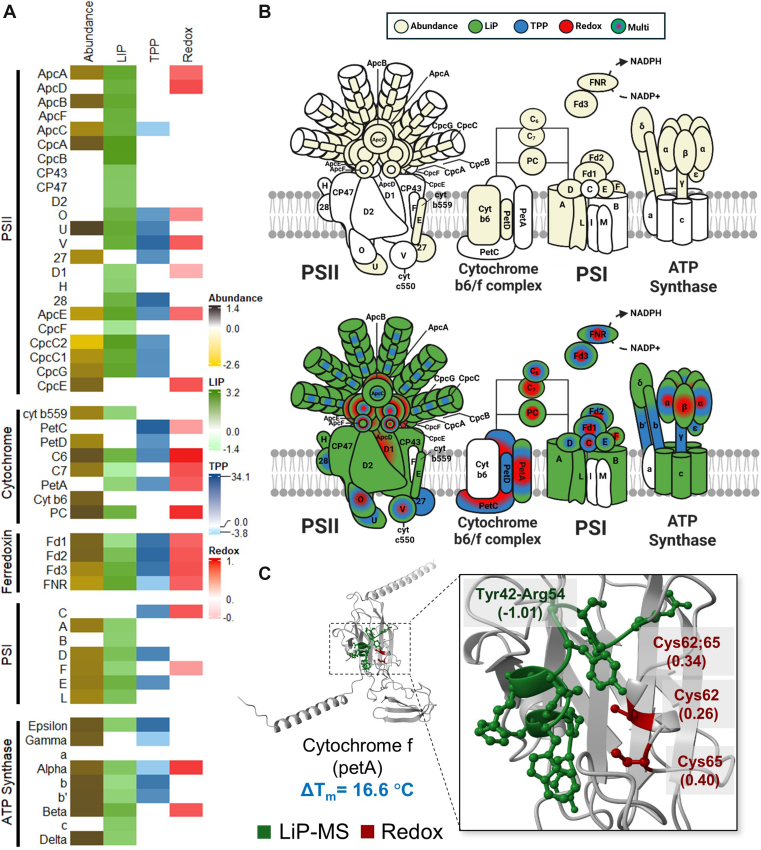
**Abundance and structural changes in cyanobacterial light-harvesting and electron transport proteins under increased light, as revealed by global proteomics, LiP, TPP, and redox profiling.***A*, heat map of proteins identified by abundance, LiP, TPP, and redox profiling. Abundance, LiP, and redox profiling values are presented on the log scale, and TPP values are presented as ΔT_m_. *B*, graphical representation of PSII, the cytochrome b6/f complex, PSI, and ATP synthase. Proteins identified as undergoing a significant change in absorbance in the dense/dilute condition are colored *yellow* (*top panel*). Proteins undergoing structural perturbations are colored *green*, *blue*, and *red*, representing the identifying technique or a gradient if identified by multiple techniques (LiP, TPP, and redox profiling respectively, *bottom panel*). *D*, cytochrome f (petA) was found to be significantly affected by LiP, TPP, and redox proteomics but not by traditional proteomics. According to TPP, the melting point increased by 16.6 °C in the dilute culture, indicating the protein structure is more stable. The statistically significant semi-tryptic peptides (from LiP data) and cysteine residues (from redox proteomics data), and their respective log_2_fold changes are indicated on the alpha fold predicted structure of the protein. LiP, limited proteolysis; TPP, thermal proteome profiling; PSII, photosystem II; PSI, photosystem I.

Recognizing that many of the altered proteins identified were integral components of key cellular complexes, we next conducted a comparative analysis on specific macromolecular assemblies impacted by increased light exposure. For this analysis, we focused on photosynthetic complexes only on the single perturbation, as it resulted in the most robust experimental changes in *S. elongatus*. Notably, this pathway only appears to be enriched in the LiP-MS dataset alone. However, a deeper examination revealed contributions from the other structural proteomics technologies when analyzing individual protein fluctuations. These are exemplified in a heatmap summarizing changes across light-harvesting and electron transport chain complexes based on fold changes in abundance from traditional global proteomics analysis, perturbation scores from LiP-MS, melting temperature changes from TPP-MS, and thiol oxidation fold changes from redox proteomics (Fig. 8*A*). The uniport identifiers for individual proteins shown in the heatmap are provided in Supplemental Table S8. The heatmap revealed that over 1/3rd of the proteins exhibited no detectable changes in protein abundance, with structural techniques filling in a majority of the remaining gaps. For example, three of the proteins were found to be altered using LiP-MS, TPP-MS, and redox proteomics, but had no detectable changes in abundance, while only one unique protein (Cyt b6) was exclusively detected through abundance-based approaches.

To further illustrate the complementary patterns, we mapped affected proteins onto KEGG pathways of the photosynthetic light-harvesting and electron transport chain complexes (Fig. 8*B*). In the top panel, changes in protein abundance alone were visualized and shaded in yellow. Several key proteins within the complexes were clearly detected using traditional proteomics. However, the bottom panel, which includes contributions exclusively from the structural proteomics approaches, revealed a striking cluster of proteins localized primarily in intra-membrane regions. These findings emphasize the distinct ability of the structural techniques to identify functionally relevant protein changes in regions often inaccessible to traditional abundance measurement.

We further honed-in on cytochrome f protein (PetA), which was significantly altered by LiP-MS, TPP-MS, and redox proteomics. Using AlphaFold, we generated a predicted structure of PetA and mapped changes observed in semi-tryptic peptides from LiP-MS alongside oxidized Cys residues identified through redox proteomics (Fig. 8*C*). Semi-tryptic peptides spanning Tyr42-Arg54 showed reduced solvent accessibility, indicative of conformational remodeling in this region. Spatially proximal to this region were two oxidized Cys residues (Cys62 and Cys65) identified across multiple peptide-spectrum matches. These observations suggest that the two Cys residues may form a disulfide bridge under dilute conditions with higher light exposure, potentially stabilized by the aromatic residues present in the conformationally altered region identified by LiP-MS. Such an interaction would likely result in a more compact and stable structure, corroborated by the positive ΔT_m_ in the TPP-MS analysis. This example showcases how structural proteomics not only fills gaps in functional pathway data but also provides deeper insights into conformational changes related to PTMs that potentially modulate protein function within a pathway in response to environmental cues.

The ribosome complex and associated translation pathways were most significantly impacted after increased light exposure, aligning with previous findings in *S. elongatus* (48). Comparative analysis of changes in the complex detected by the different techniques revealed substantial proteomics changes. Heatmaps show that the majority of the proteomic changes were affected at the level of global abundance changes (Supplemental Fig. S7*A*). The structural techniques provided additional insights into ribosomal adaptation, with LiP-MS showing the widest coverage and TPP capturing temperature increases across many of the subunits. Taken together with increased abundance measures, these thermal shifts are consistent with increased ribosome synthesis and assembly likely associated with heightened protein synthesis demands under the high-light condition. Fewer subunits exhibited changes in oxidation states with those exhibiting changes likely driven by oxidative stress due to increased ROS rather than direct redox regulation of ribosome function.

Mapping abundance and structural changes onto KEGG’s ribosome pathway map (03,010) again highlighted the complementarity of the methodologies (Supplemental Fig. S7*B*). While global abundance changes captured a large number of proteomic changes, structural techniques helped fill in the gaps by identifying several subunits not detectable from traditional proteomics techniques. Although clustering patterns in altered ribosomal subunits were less pronounced than those observed in the phycobilisome membrane proteins, oxidation-specific changes appeared to concentrate in particular subunit regions, suggesting that certain areas of the ribosome may be more susceptible to ROS-driven impacts.

## Discussion

Phototrophs, such as cyanobacteria, face significant challenges related to light availability and distribution in both natural ecosystems and industrial bioproduction systems, where dense cultures lead to self-shading and heterogeneous light conditions (49, 50). In agreement with previous studies, our structural proteomics tools revealed how these fluctuations trigger molecular remodeling of photosynthetic machinery (51, 52, 53), protein synthesis factories (54, 55, 56, 57, 58), and central carbon metabolism pathways (59, 60, 61), enabling cyanobacteria to rapidly shift between energy storage and utilization. Our complementary tools provided functional insights beyond those captured by traditional abundance-based approaches, as demonstrated in the transient dark treatment, where only 145 proteins showed abundance changes compared to over 400 proteins that showed structural modifications. These findings indicate that rapid protein conformational changes, rather than abundance shifts, serve as critical drivers of cyanobacteria adaptation under fluctuating light conditions, offering deeper mechanistic insights into microbial phenotypic responses.

The limited overlap (<10%) among the proteins identified as significant across the global, LiP, TPP, and redox proteomics datasets reflects the distinct chemical and biophysical features measured by each technique. In particular, we observed that the lack of overlap was largely driven by differences between TPP and redox proteomics, suggesting that Cys oxidation, while detectable through redox proteomics, may not induce a sufficient structural change to alter protein thermal stability. While we initially anticipated greater overlap across these methods, the complementary insights provided by each technique proved to be more informative for understanding distinct aspects of individual protein responses.

Interestingly, our redox proteomics findings in this study, which show increased Cys oxidation under high light exposure, differ from some prior studies, such as Guo *et al.* (30), that reported a more reduced proteome under light conditions. These discrepancies likely stem from methodological differences, such as the inability of current redox proteomics techniques to distinguish between reversible disulfide bonds and irreversible oxidative modifications, as well as biological factors such as differences in light intensity between studies. Guo *et al*. used more moderate light conditions, generating reducing power *via* the ferredoxin-thioredoxin system, driving Cys residues to more reduced states with reversible disulfide formation (42, 43). In contrast, our study and others (62) used high light intensities, which induces photo-oxidative stress, generating ROS that cause irreversible Cys oxidation and override reductive pathways (39, 40).

We observed the most robust molecular changes upon dilution, where the cyanobacteria transitioned from a light-limited, self-shading state to light enhanced light exposure—as experienced by cells in bioreactor cores, pond interiors, or surface waters (49, 50). Thirty minutes of enhanced light exposure triggered significant remodeling of photosynthetic machinery (Fig. 8), consistent with phototrophic adaptation strategies that prioritize optimizing light harvesting machinery and mitigating associated stress responses (18, 63). Regulation of light harvesting begins with the phycobilisome, a light-harvesting antenna that transfers energy to photosystem II (PSII) and, to a lesser degree, photosystem I (PSI) complexes (64). Structural reorganization of PBS rods, comprised of C-phycocyanin (Cpc) subunits (65, 66) and cores, formed by allophycocyanin subunits (67) were evident from both structural and abundance changes. Increased melting temperatures suggested stabilization of the rods in response to high-light exposure (65, 66, 67) despite high-light conditions typically inducing PBS dissociation to protect photosynthetic machinery. Our study suggests full disassembly of the PBS may not have occurred within our 30-min exposure period. Notably, NblA, a known marker of PBS degradation (68, 69) was not changed in our dataset, suggesting the PBS stayed intact, though they may have been dissociated from the photosystem assembles, which can occur on the order of picoseconds (70). Supporting this notion, we observed that HspA, a molecular chaperone known to stabilize PBS subunits under light-induced oxidative stress (71, 72, 73, 74) was significantly more abundant, suggesting that HspA-mediated stabilization plays a key role in maintaining light-harvesting function during early light stress.

The PBS complex coordinates energy through direct interactions with PSI and PSII, forming supercomplexes at the membrane surface (75, 76, 77). While abundance changes in the PSI and PSII subunits were minimal, structural proteomics technologies revealed major conformational changes in the protein subunits consistent with reports of short-term structural state transitions of PSII between light-harvesting and energy-quenching states (78, 79). In both the PBS and photosystem assemblies, we observed dynamic conformational and redox changes, aligning with known regulatory interactions between PBS components and membrane-localized photosystem *via* redox signaling pathways (18, 80).

Beyond the PBS and photosystems, we observed adaptations in the cytochrome *b6/f* complex, particularly in cytochrome *f* (PetA), a rate-limiting component of linear electron flow between PSII and PSI. PetA displayed no changes in abundance but showed significant structural modifications, including increased thermal stability (ΔT_m_=16.6 °C, TPP), reduced solvent accessibility (Tyr42–Arg54, log_2_FC = −1.01, LiP-MS), and increases in Cys oxidation (Cys62/65, log_2_FC = 0.25–0.39, redox proteomics) (Fig. 8*C*). These coordinated structural and redox modifications align with prior evidence linking PetA stability to high-light acclimation and enhanced electron transport efficiency (22, 81). This ability of cyanobacteria to toggle PetA structure to fine-tune electron transport exemplifies the power of the structural proteomics technologies in revealing otherwise unseen molecular mechanisms that are triggered in response to fluctuating light conditions.

Cyanobacterial responses to increased light exposure extended beyond photosynthetic machinery to protein synthesis regulation, as evidenced by structural adaptations in ribosomal proteins detected by all proteomic technologies (Fig. 6 and Supplemental Fig. S5). Despite observations of the ribosome pathway enrichment observed in traditional abundance workflows, its appearance in LiP-MS and TPP-MS datasets points to structural adjustments beyond abundance changes. Known to act as circadian clock-controlled regulatory points aligning protein synthesis with light cycles (82, 83), our data indicates that ribosomes also function as environmental sensors, suggesting coordinated responses to light energy availability. Similar dynamic ribosomal modifications have been reported in heterotrophic organisms (57, 84, 85), though precise spatial relationships require higher-resolution platforms such as cryo-EM for validation. These structural states likely link ribosomal assembly and activity to stringent responses, hibernation pathways, and translation control mechanisms that synchronize cellular growth and energy metabolism during stress (83, 86, 87, 88).

Lastly, our findings revealed extensive remodeling of key enzymes within central carbon metabolism triggered by fluctuating light conditions. We observed structural changes in major pathways, including the Calvin-Benson-Bassham cycle, TCA cycle, pentose phosphate pathway, and glycolysis (Fig. 7). Key proteins such as RuBisCO and CcmM presented consistent structural modifications across all technologies, highlighting the coordinated regulation of carbon fixation under stress. Structural changes observed in CcmM (Fig. 3), a scaffolding protein central to carboxysome assembly, align with prior reports that carboxysome flexibility facilitates rapid environmental adaptation by optimizing carbon metabolism (38). We observed significant structural perturbations for other carboxysome core proteins CcmK2, CcmK4, CcmL, and CcmO *via* LiP and TPP (Supplemental Tables S4 and S5), further supporting increased carboxysome dynamics in response to environmental signals. RuBisCO exhibited increased T_m_ and structural modifications consistent with dynamic interactions with CcmM (89), further connecting light-induced adaptations in photosynthetic machinery to carbon fixation processes (38, 90). These findings reinforce the complementary strengths of the structural techniques, capturing coordinated adjustments that link photosynthesis and carbon metabolism under fluctuating stress conditions.

## Limitations and Future Directions

Our study integrates four distinct proteomic approaches to explore the molecular responses of cyanobacterial cultures to differential light availability. As with traditional global proteomics studies, the data generated here are best utilized to formulate mechanistic and testable hypotheses rather than definitive conclusions. For example, while the conformational changes identified using LiP-MS provide valuable insights into responsive assemblies, some changes observed may result from transient protein interactions or local dynamics unrelated to the perturbation. Follow-up studies using orthogonal, high-resolution approaches such as cryo-EM or X-ray crystallography, as well as targeted experiments like gene manipulation, will be crucial for further resolving these mechanisms at the structural level. Though beyond the scope of the current study, alternative experimental designs—including time-course analysis of diluted cultures under darkness or systematic variation of light intensity at constant density—would provide additional insights into the nuanced mechanisms underlying dynamic proteome regulation and how cells optimize function across a continuum of light intensities and cell densities.

Finally, we recognize the challenges in interpreting the balance between reductive and oxidative processes in cyanobacteria. At the high light intensities used in this study, ROS generation may override the reductive effects of photosynthetic activity, leading to oxidative stress responses that dominate the proteome. Conflicting results with previous reports highlight the context-dependent and complex nature of cyanobacterial redox regulation and underscore the need for standardizing experimental conditions. Expanding investigations across diverse strains and environments will help clarify whether these redox trends and proteomics responses are broadly conserved. Additionally, developing methods to distinguish reversible *versus* irreversible, we note that future development of methods to distinguish reversible versus irreversible Cys modifications would significantly advance our understanding of light-dependent redox regulation and enable more accurate comparisons across different studies and physiological conditions.

## Conclusion

In summary, structural proteomics technologies identified redox-sensitive and structurally dynamic proteins across photosynthesis, electron transport, and carbon fixation pathways, highlighting the modular adaptations that enable cyanobacteria to thrive under fluctuating environmental conditions. By revealing the coordinated regulation of PTMs and protein conformational changes across sequential metabolic steps, these tools provide deeper insights into microbial adaptability and potential engineering targets. Further studies with finer temporal resolution and extended environmental stress paradigms could refine these observations, while integration with metabolomics, flux analysis, and protein–protein interaction studies would establish causal links between molecular remodeling and phenotypic outcomes. Together, these approaches could build predictive models for microbial phenotypes, transforming both fundamental understanding of cyanobacterial adaptation and practical strain engineering for industrial applications.

## Data availability

Primary liquid chromatography-mass spectrometry raw measurement data are openly accessible for download at the Mass Spectrometry Interactive Virtual Environment (MassIVE) community repository under the following accession URIs: https://identifiers.org/massive:MSV000098675 (LiP-MS-Opt), https://identifiers.org/massive:MSV000098676 (LiP-MS-Pro), and https://identifiers.org/massive:MSV000098679 (TPP-MS-TMT10). Processed proteome datasets and supporting metadata files are openly accessible for download at the PNNL DataHub Predictive Phenomics Initiative (PPI) project data package DOI: PPI/2574396" title = "https://doi.org/10.25584/PPI/2574396">https://doi.org/10.25584/PPI/2574396. Dataset download includes a sample naming key, normalized quantification data, computed outputs, and protein-annotated results files. All corresponding dataset accessions, including previously reported raw measurement data (“Redox-MS” MC-DP2 data package) (91), and related software supporting analysis transparency and reuse have been provided at the DataHub dataset DOI download page.

The source code workflow described here is openly available from the Predictive Phenomics Initiative GitHub repository under “Microbial-Isolate-LiP-Analysis” (https://github.com/PNNL-Predictive-Phenomics/Microbial-Isolate-LiP-Analysis), supporting data processing transparency. This workflow can be formally cited under the Zenodo DOI: https://doi.org/10.5281/zenodo.15881600 in alignment with source code community best practices for reuse.

## Supplemental data

This article contains supplemental data.

## Conflict of interest

The authors declare no competing interests.
