# Supplementary material for: Rapid Adaptation of Cyanobacteria to Environmental Perturbations Is Achieved Through Structural Remodeling of the Proteome
Source: Mol Cell Proteomics. 2025 Nov 3;25(7):101443. doi: 10.1016/j.mcpro.2025.101443 (PMC13424402; doi:10.1016/j.mcpro.2025.101443)
Supplement: Supplemental Figures [file mmc1.docx]

**Supplementary Information**

**Rapid adaptation of cyanobacteria to environmental perturbations is achieved through structural remodeling of the proteome**

Snigdha Sarkar^1,#^, Elise M. Van Fossen^1,#^, Xiaolu Li^1^, Tong Zhang^1^, Song Feng^1^, Victoria Prozapas^1^, Ivo Díaz Ludovico^1^, Abdullah D. Shouaib^2^, Chelsea M. Hutchinson-Bunch^1^, Natalie Sadler^1^, Isaac K. Attah^1^, Wei-Jun Qian^1^, Margaret S. Cheung^3,4^, Pavlo Bohutskyi^1,5,*^, John T. Melchior^1, 6, 7*^

^1^Biological Sciences Division, Earth and Biological Sciences Directorate, Pacific Northwest National Laboratory, Richland, WA, USA.

^2^Nuclear Chem Bio Technologies, National Security Directorate, Pacific Northwest National Laboratory, Richland, WA, USA.

^3^Environmental Molecular Sciences Laboratory, Earth and Biological Sciences Directorate, Pacific Northwest National Laboratory, Richland, WA, USA.

^4^Department of Physics, University of Washington, Seattle, WA, USA

^5^Department of Biological Systems Engineering, Washington State University, Pullman, WA, USA

^6^Department of Pathology and Laboratory Medicine, University of Cincinnati, Cincinnati, OH 45237

^7^Department of Neurology, Oregon Health and Science University, Portland, Oregon 97239 USA

# Authors contributed equally

* To whom correspondence should be addressed:

Pavlo Bohutskyi: E-mail: [pavlo.bohutskyi@pnnl.gov](mailto:pavlo.bohutskyi@pnnl.gov)

John Melchior: E-mail: [john.melchior@pnnl.gov](mailto:john.melchior@pnnl.gov)

**Figures**

**Supporting figure S1.** Initial assessment of overall proteome stability with a wide temperature range.

**Supporting figure S2.** Annotated MS/MS spectra.

**Supporting figure S3.** Relationship between the Perturbation score, solvent accessibility score, and protein length.

**Supporting figure S4.** Distribution of the R^2^ values of fitted melting curves in TPP experiment sets.

**Supporting figure S5.** Functional enrichment analysis with proteins classified in LiP-MS structural levels.

**Supporting figure S6.** UniProt IDs of carbon metabolism genes.

**Supporting figure S7.** Abundance changes and structural alterations of ribosomal complexes in cyanobacteria exposed to increased light as determined by global proteomics, LiP, TPP and redox profiling.


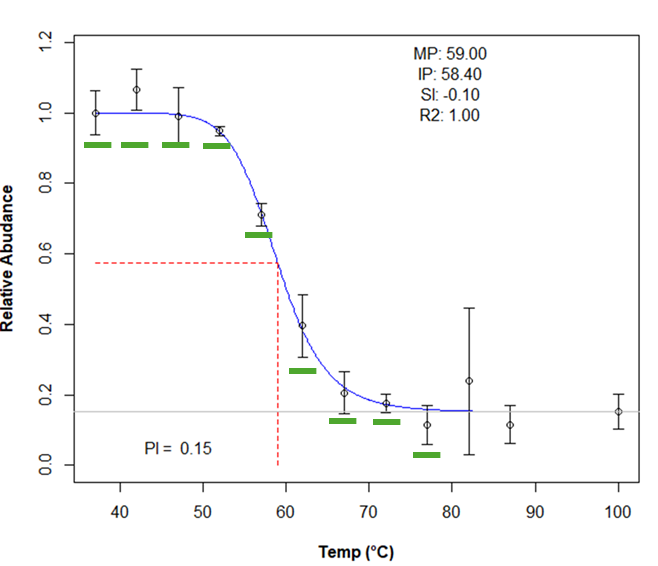


**Supporting figure S1. Initial assessment of overall proteome stability with a wide temperature range.** Final ten selected temperatures are indicated by green lines.


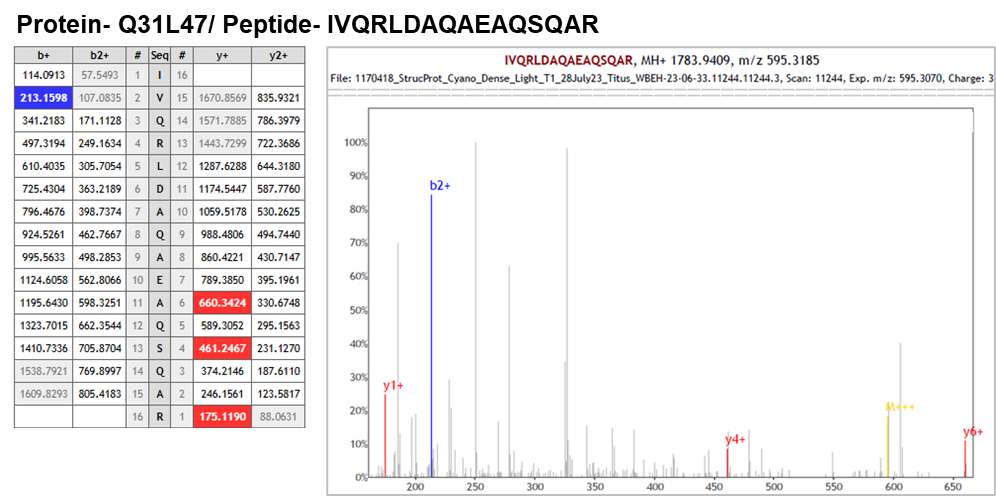

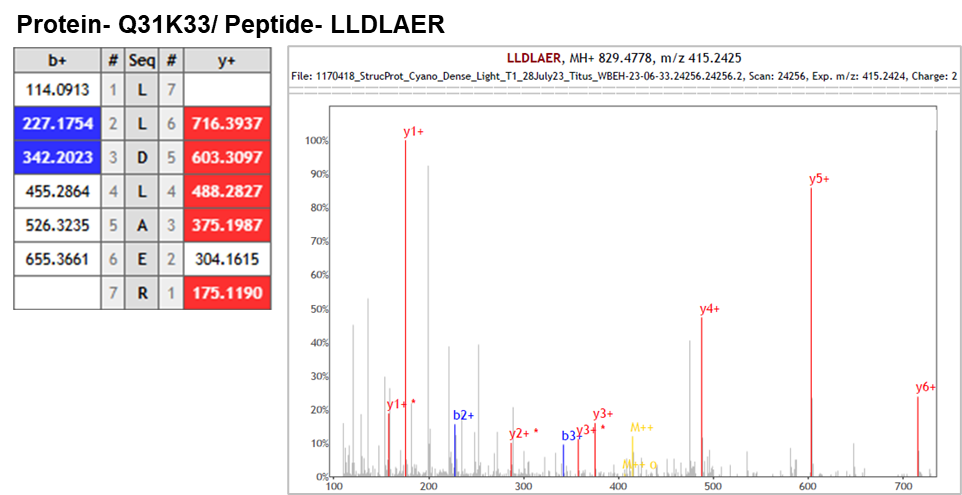


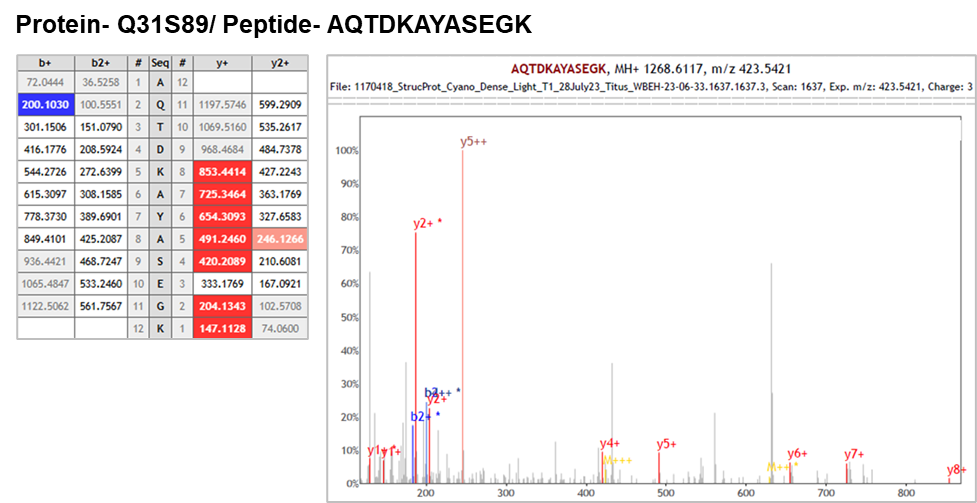

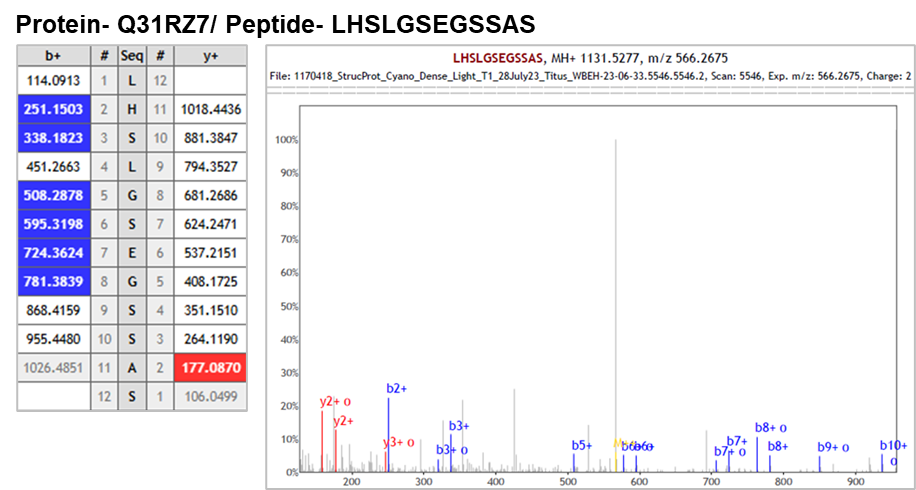


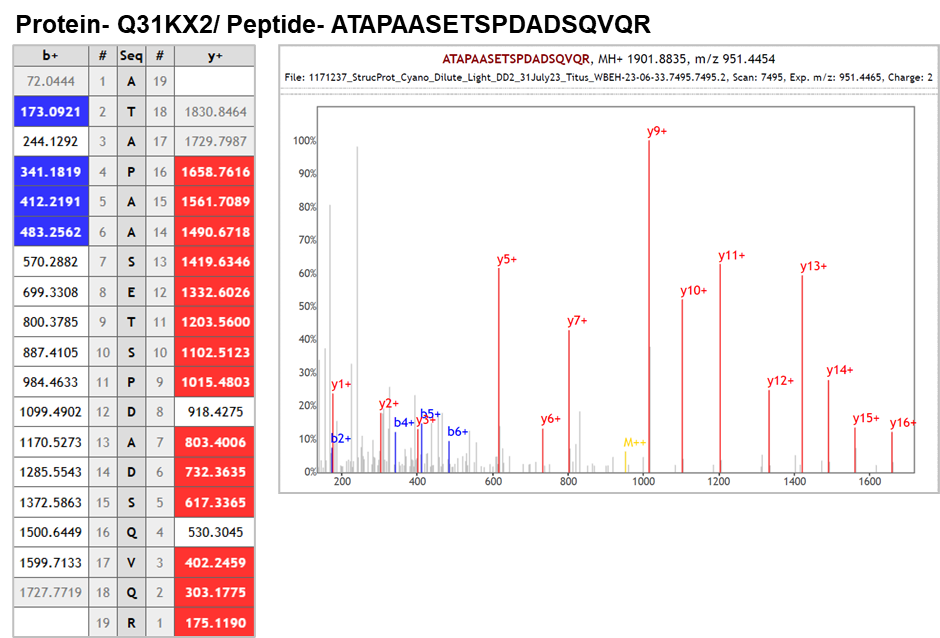

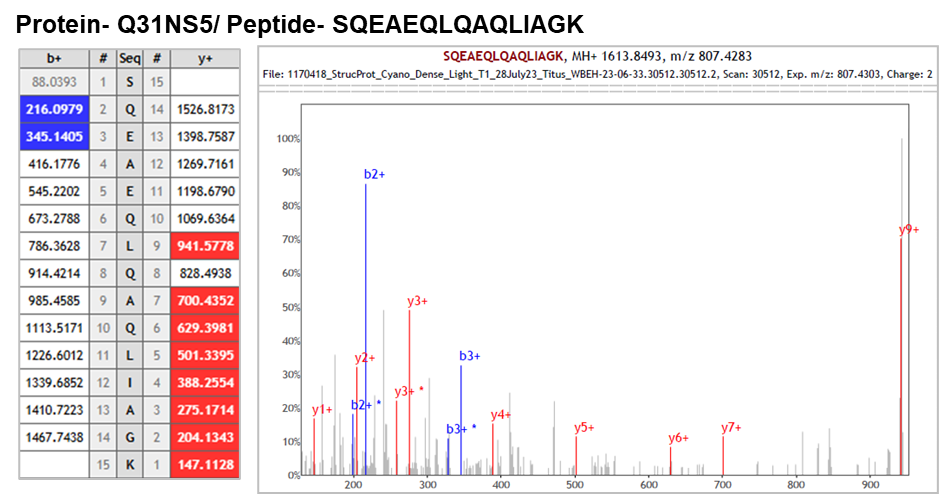


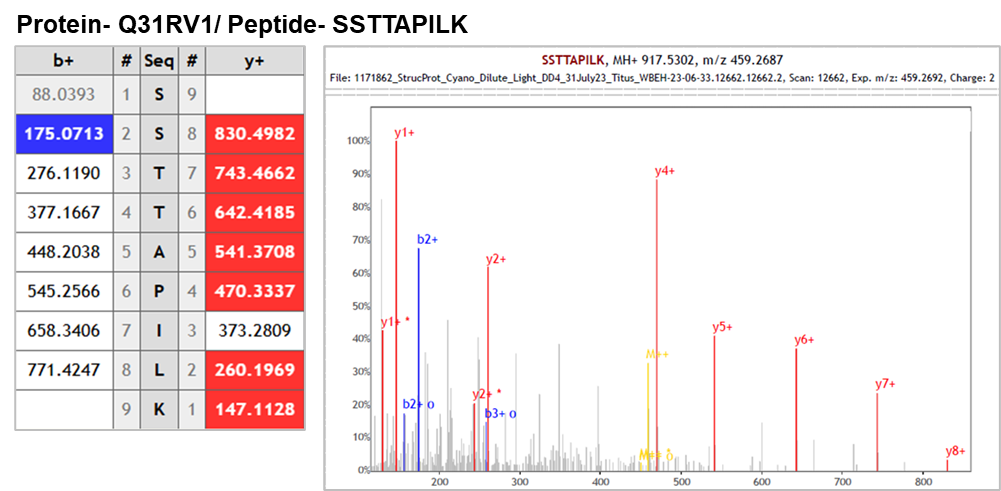


**Supporting figure S2. Annotated MS/MS spectra.** A small number of proteins that were identified on the basis of a single unique peptide were left after data processing. The annotated LC-MS/MS spectra for the unique peptides from these proteins are shown here.


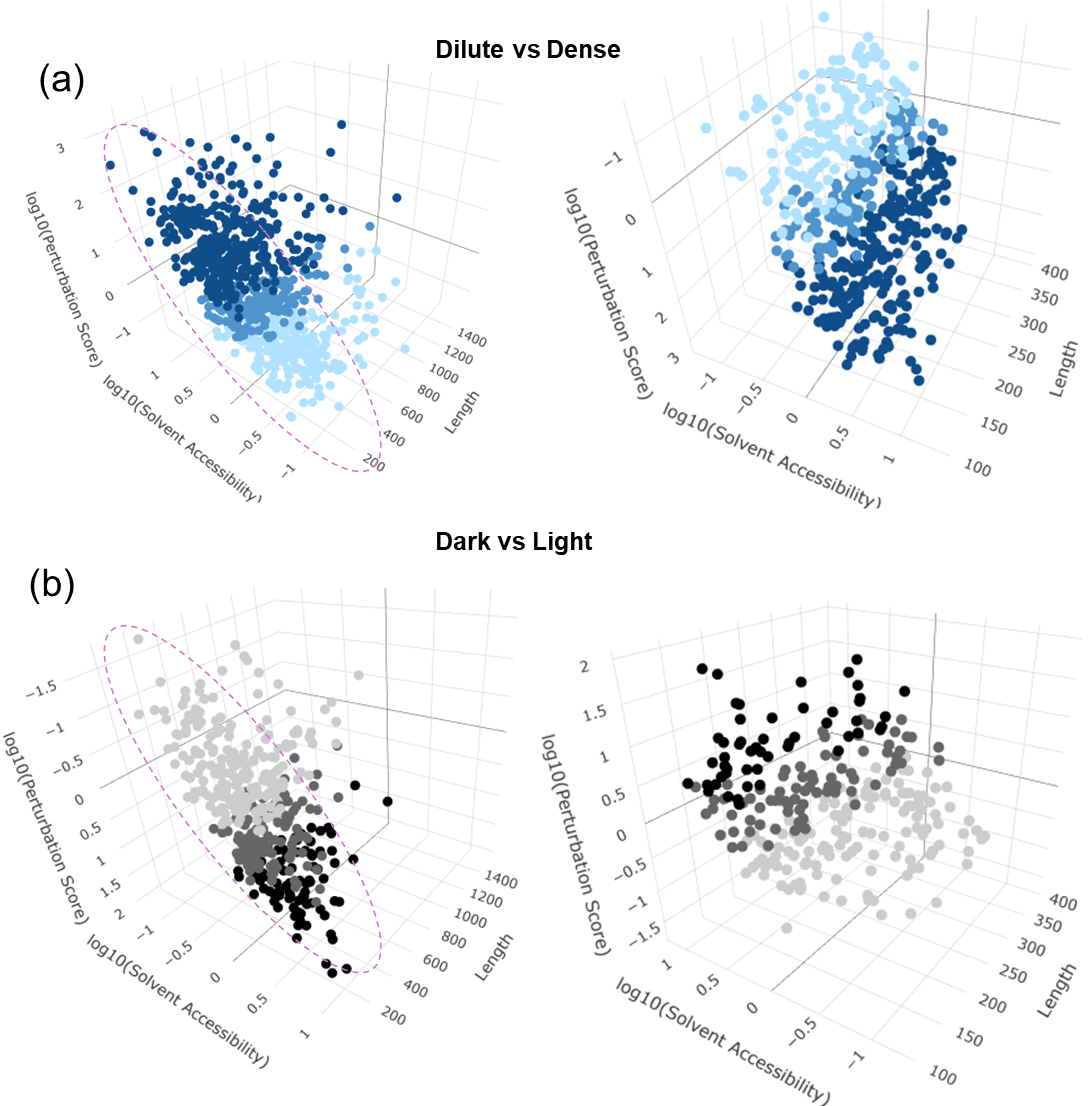


**Supporting figure S3. Relationship between the Perturbation score, solvent accessibility score, and protein length**. A 3D-scatter plot between the three parameters for (a) dense vs dilute, and (b) dark vs light comparisons show that neither the perturbation score nor the solvent accessibility score are biased by protein length.


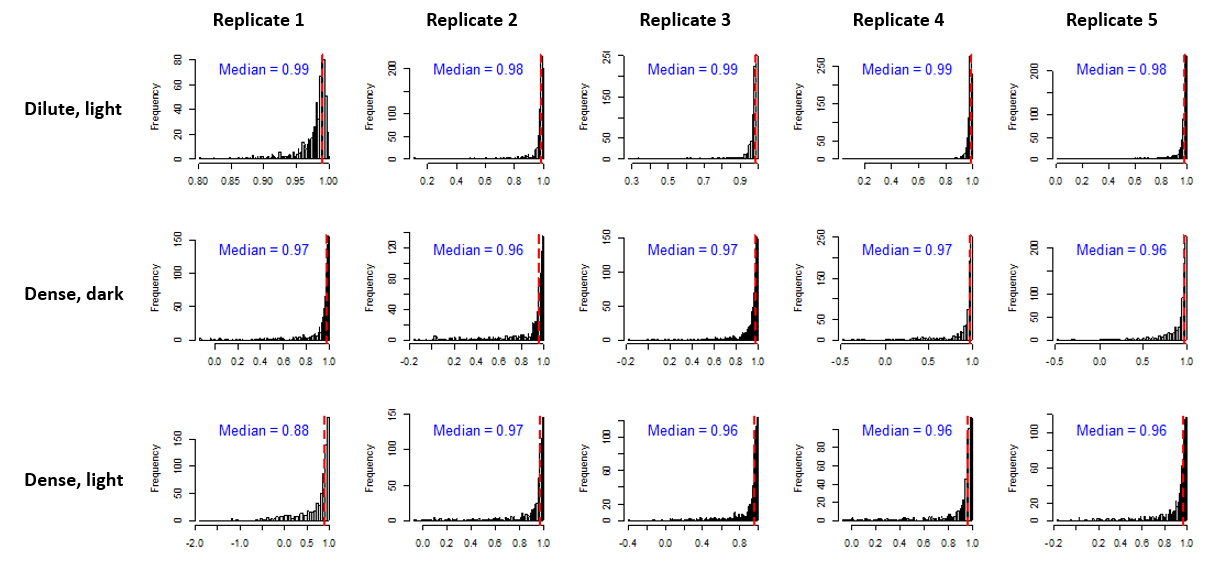


**Supporting figure S4.** Distribution of the R2 values of fitted melting curves in TPP experiment sets.


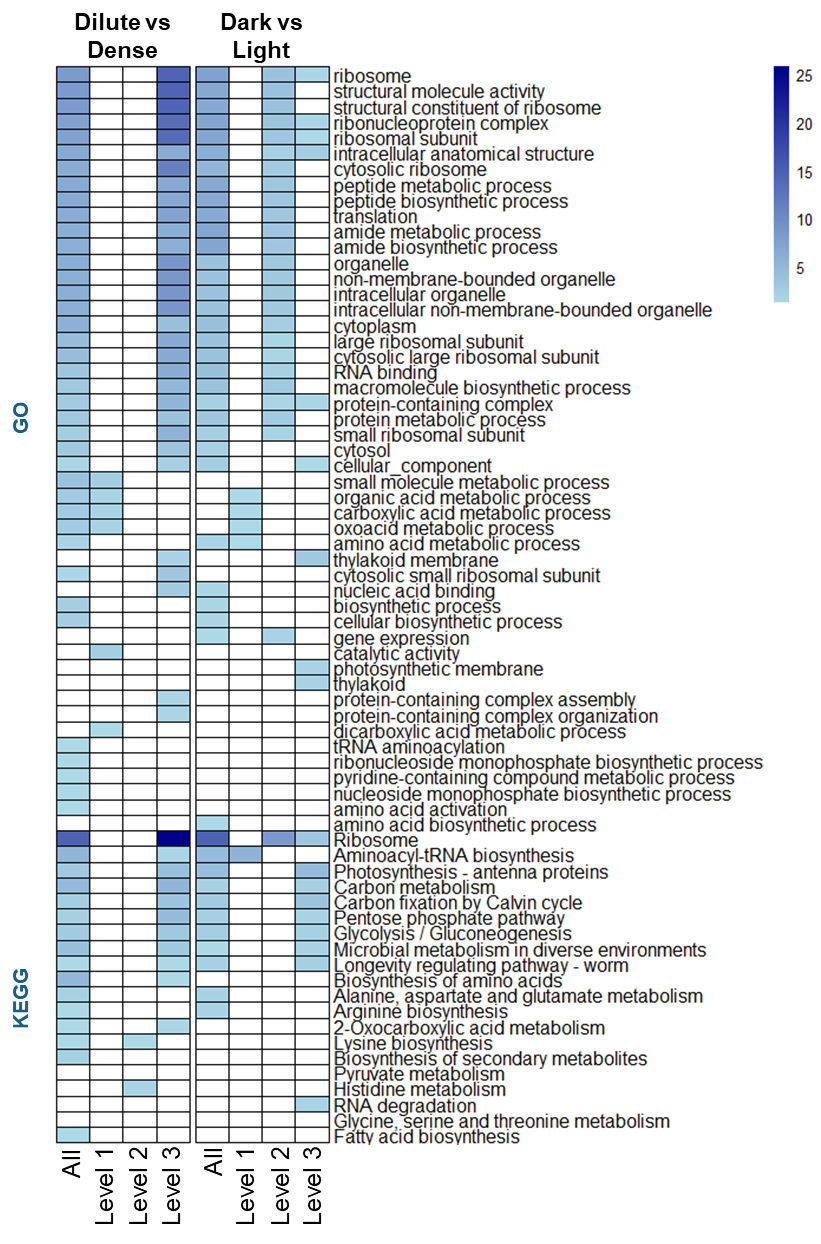


-log_10_(p_adjusted_)

**Supporting figure S5.** Functional enrichment analysis with proteins classified in LiP-MS structural levels.


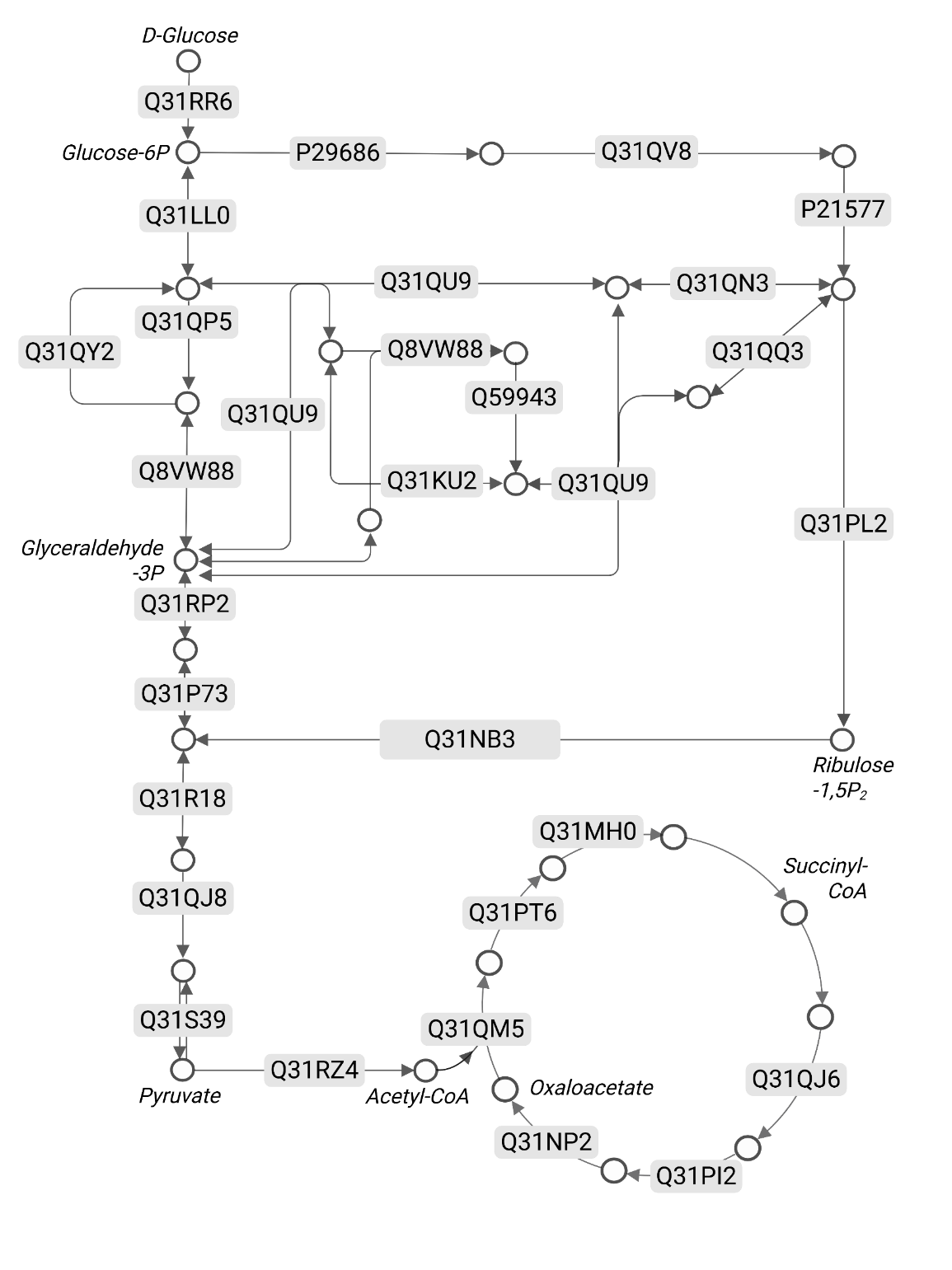


**Supporting figure S6. UniProt IDs of carbon metabolism genes.** The metabolic network is adapted from the KEGG carbon metabolism pathway (syf01200). The UniProt identifiers for the enzymes catalyzing each metabolic reaction is annotated along the pathway.


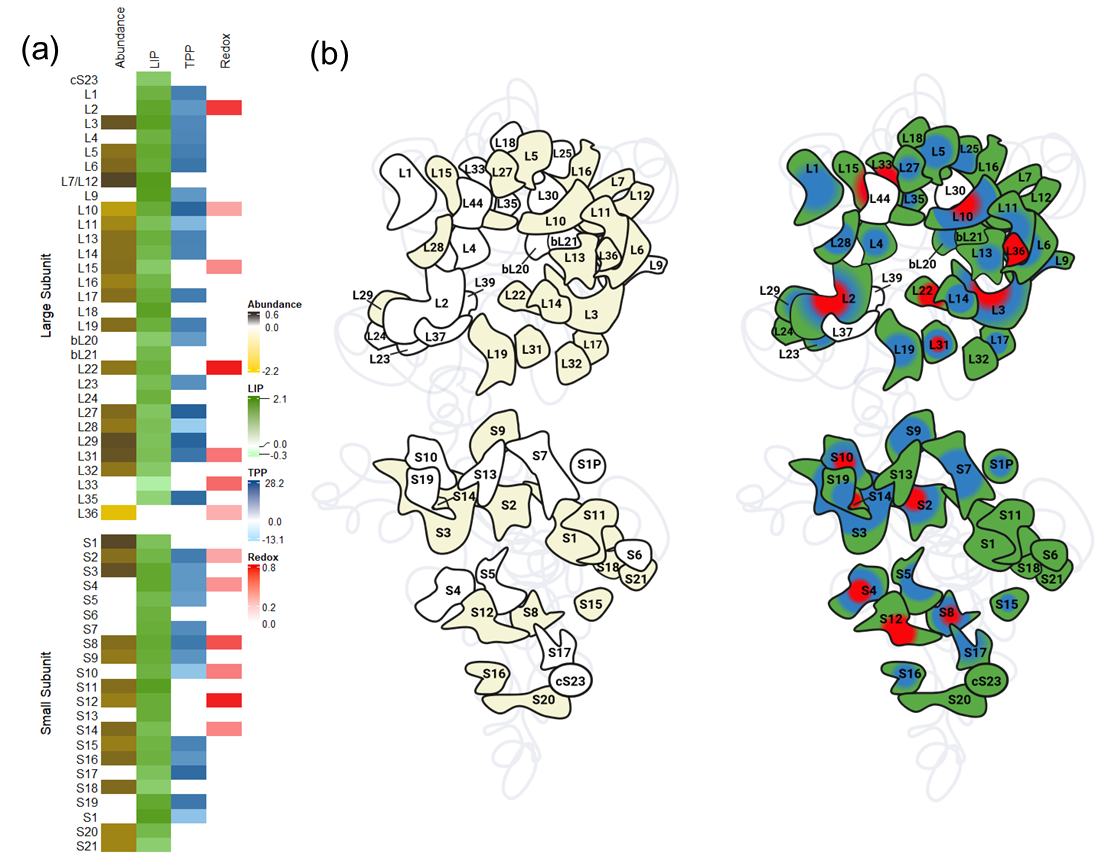


**Abundance**

**TPP**

**Multi**

**LiP**

**Redox**

**Supporting figure S7. Abundance changes and structural alterations of ribosomal complexes in cyanobacteria exposed to increased light as determined by global proteomics, LiP, TPP and redox profiling.** A) Heat map of proteins identified by abundance, LiP, TPP and redox profiling. Abundance, LiP and redox profiling values are presented as log_10_, TPP values are presented as ΔT_M_ (B) Graphical representation of the large and small ribosomal complexes. Proteins identified as undergoing a significant change in absorbance in the dense/dilute condition are colored yellow (top panel). Proteins undergoing structural perturbations are colored green, blue and red representing the identifying technique, or a gradient if identified by multiple techniques (LiP, TPP and redox profiling respectively, bottom panel).
